# Affinity Proteomics‐Based Non‐Invasive Detection of Clinically Significant Liver Disease

**DOI:** 10.1111/apt.70656

**Published:** 2026-04-16

**Authors:** Sriram Balasubramani, Katharina Remih, Anna Sophie Karl, Julia Alexandra Borchert, Christina Schrader, Malin Fromme, Can Kayatekin, Bailin Zhang, Mikhail Levit, Pavithra Krishnaswami, Louise E. van Eekeren, Leo A. B. Joosten, Twan Otten, Petra Tomanová, Pavel Strnad

**Affiliations:** ^1^ Medical Clinic III, Gastroenterology, Metabolic Diseases and Intensive Care Health Care Provider of the European Reference Network on Rare Liver Disorders (ERN RARE LIVER), University Hospital RWTH Aachen Aachen Germany; ^2^ Department of Gastroenterology and Hepatology Medical University Lausitz‐Carl‐Thiem Cottbus Germany; ^3^ Sanofi Cambridge Massachusetts USA; ^4^ Department of Internal Medicine Radboud University Medical Centre Nijmegen the Netherlands; ^5^ Department of Medical Genetics Iuliu Hatieganu University of Medicine and Pharmacy Cluj‐Napoca Romania; ^6^ Prague University of Economics and Business Prague Czech Republic

**Keywords:** alpha‐1 antitrypsin deficiency, non‐invasive liver test, PEA, plasma proteomics, UK Biobank

## Abstract

**Background:**

Non‐invasive biomarkers predicting major adverse liver outcomes (MALO) are urgently needed.

**Aims:**

We assessed a novel, proximity extension assay‐based high‐throughput targeted proteomics.

**Methods:**

Plasma proteomic data (> 2900 proteins) and clinical information were accessed from the population‐based UK Biobank cohort, comprising ~52,000 individuals with a median follow‐up of > 10 years, including obese (~12,700) and diabetic (~1500) participants. Validation cohorts comprised 287 participants with severe alpha1‐antitrypsin deficiency (Pi*ZZ genotype), and 960 people living with HIV, who underwent liver stiffness measurement (LSM) via transient elastography. Selected proteomic parameters were compared to routine measurements. Bayes‐moderated linear models (covariates: age and sex) assessed the differential abundance. Logistic regression was used to develop and validate a prognostic score.

**Results:**

Routine gamma‐glutamyltransferase (GGT) and aspartate aminotransferase (AST) strongly correlated with proteomic measurements (*r* = 0.92 and *r* = 0.71, respectively). Similarly, proteomic‐based thrombospondin‐2 levels correlated with immunoassay‐based values (*r* = 0.85). Twenty proteins were consistently associated with future MALOs/increased liver stiffness in all cohorts. A novel five‐component proteomic score derived from the UK Biobank cohort demonstrated superior predictive power for MALOs (AUROC = 0.84) compared to AST‐to‐platelet‐ratio index (AUROC = 0.73) and Fibrosis‐4 index (AUROC = 0.72), with stable performance in obese and diabetic subcohorts. In people living with HIV and alpha1‐antitrypsin deficiency patients, all five components increased across fibrosis stages and the proteomic score outperformed AST‐to‐platelet‐ratio/Fibrosis‐4 index in predicting significant LSM‐based liver fibrosis.

**Conclusions:**

We identify a new proteomic score comprising epithelial/hepatic stellate cell markers that yields a robust predictive performance across different liver disease aetiologies. Thereby, we demonstrate the usefulness of emerging proteomic techniques in hepatology.

**Trial Registration:**

The registry study is listed at ClinicalTrials.gov (NCT02929940)

## Graphical abstract

Using UK Biobank proteomic data, we identified a five‐protein score reflecting hepatic stellate cell activation and hepatocellular injury that predicts major adverse liver outcomes and clinically significant fibrosis, with consistent performance validated in two independent cohorts (patients with HIV and alpha1‐antitrypsin deficiency).

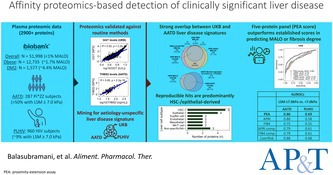

**Abbreviations**
AATD: alpha1‐antitrypsin deficiency
ADAMTSL2: ADAMTS‐like protein 2
ALDH1A1: aldehyde dehydrogenase 1A1
ALT: alanine aminotransferase
APRI: AST‐to‐platelet‐ratio index
AST: aspartate aminotransferase
AUROC: area under receiver‐operating curve
DM2: type‐2 diabetes
FDR: false discovery rate
FIB4: fibrosis‐4 index
GGT: gamma‐glutamyltransferase
HSC: hepatic stellate cell
ICD‐10: International Classification of Diseases version 10
IGFBP7: insulin‐like growth factor‐binding protein 7
ITGBL1: integrin beta‐like protein 1
KRT18: keratin‐18
LSM: liver stiffness measurement
MALO: major adverse liver outcomes
MENT: C1orf56 (chromosome 1 open reading frame 56)
NPX: normalised protein expression
OPCS‐4: operations/procedure codes version 4
PEA: proximity extension assay
Pi*ZZ: homozygous Pi*Z mutation in alpha1‐antitrypsin gene
PLHIV: people living with HIV
TE: transient elastography
THBS2: thrombospondin‐2
UKB: the UK Biobank cohort

## 1 Introduction

Although potentially preventable, liver‐related mortality constitutes the second leading cause of lost working life in Europe [1]. Furthermore, liver disease is often asymptomatic and becomes only apparent when major adverse liver outcomes (MALO) such as ascites, variceal bleeding or hepatocellular carcinoma occur. However, such a late diagnosis is associated with poor prognosis [1]. Therefore, screening for liver disease is of utmost importance, especially in participants at increased risk, such as obese individuals or patients with diabetes [2]. Serum liver enzymes, that is, aspartate/alanine aminotransferase (AST/ALT) or gamma‐glutamyltransferase (GGT), are commonly used; however, they reflect short‐term damage rather than the long‐term remodelling known as fibrosis that is of key prognostic relevance [1, 3]. For the latter, several serum marker‐based scores, such as fibrosis‐4 (FIB4), have been developed that often combine liver injury markers with platelet count as a marker of advanced liver fibrosis with portal hypertension [4]. Alternatively, liver stiffness measurement (LSM) via ultrasound or magnetic resonance imaging is used as a surrogate of liver scarring [5]. Since the liver represents a key secretory organ responsible for producing most of the proteins found in the bloodstream, serum/plasma proteomics constitutes an attractive and emerging approach to identifying new biomarkers [6, 7]. In addition to the traditional, mass spectrometry‐based approach, several commercially available, affinity‐based platforms have been developed and offer both high‐throughput as well as worldwide standardisation [8, 9]. Among them, the aptamer‐based SomaScan technology and the proximity extension assay (PEA)‐based Olink platform are the most widely used [10, 11]. The latter has been extensively studied in the UK Biobank (UKB), a large‐scale community‐based, prospective cohort, where it yielded multiple valuable insights into human health [12, 13]. In contrast, the applicability of this technique in hepatology remains less well explored. To change that, we assessed the usefulness of the PEA technology to predict MALOs in the UKB cohort. We assessed the whole proteomic UKB cohort as well as subcohorts of participants with obesity and type‐2 diabetes (DM2), as established high‐risk groups that may particularly benefit from liver disease screening. The findings were validated in cohorts of patients with severe alpha1‐antitrypsin deficiency (AATD), an established proteotoxic liver disease [14, 15] and people living with HIV (PLHIV) [16, 17]. The performance of the novel PEA score was compared to values obtained with alternative techniques, including the LiverRisk score that was validated in multiple independent cohorts [18].

## 2 Materials and Methods

### 2.1 Patient Cohorts

#### 2.1.1 UKB Cohort

The UKB comprises a large‐scale, community‐based prospective study that recruited ~500,000 participants with a mean follow‐up period of 13.5 years [19]. The baseline dataset includes demographic data (age, sex, ethnicity), clinical information (BMI, comorbidities such as DM2) and laboratory values. Baseline visits took place between 2006 and 2010 in 22 centres across the UK. Inpatient hospital records starting in 1996 were used to obtain diagnoses based on the International Classification of Diseases version 10 (ICD‐10) and operations/procedures performed based on operations/procedure codes version 4 (OPCS‐4). Mortality data, including date/cause of death, were accessed through national death registries to analyse the associations between proteomic biomarkers and overall/liver‐related (primary cause of death stated with ICD‐10 codes K70–K77) mortality. All data handling and analyses were performed under the guidelines and regulations approved by the UKB, under application number 148742.

#### 2.1.2 AATD Cohort

The cohort consisted of 287 plasma samples collected from individuals with genetically confirmed homozygous Pi*Z mutation in alpha1‐antitrypsin gene (Pi*ZZ) as part of the European Alpha1 liver study [20, 21, 22]. All participants underwent thorough liver/lung phenotyping by a standardised work‐up comprising questionnaires and laboratory analyses, including standard serological testing with liver enzymes or platelet count. LSM via transient elastography [TE, (FibroScan, Echosens, Paris, France)] was performed by experienced medical staff. The examinations were conducted between 2015 and 2023. Inclusion criteria for participation were (i) age ≥ 18 years, (ii) no pregnancy and (iii) ability to provide written informed consent. Exclusion criteria were previous liver transplantation and inability to meet criteria described above. Plasma thrombospondin‐2 (THBS2) levels were quantified with an immunoassay kit (DTSP20, R&D Systems, Abingdon, UK). The Ethics Committee of Aachen University (Aachen: EK 173/15) provided ethical approval, and all participants gave written informed consent. Everyone was assessed following the ethical guidelines of the Helsinki Declaration (Hong Kong amendment) and Good Clinical Practice (European guidelines). The registry study is listed at ClinicalTrials.gov (NCT02929940).

#### 2.1.3 PLHIV Cohort

The previously published PLHIV cohort consists of 960 participants of the 2000HIV study [16, 17]. At the baseline visit, they underwent a standardised examination comprising questionnaires, blood sampling, LSM via TE and clinical data extraction from medical records. Laboratory parameters used for clinical assessments were obtained from available clinical data.

### 2.2 Definition of Patient Subgroups

Patients with MALOs were identified from UKB using a combination of ICD‐10/OPCS‐4 codes as well as cancer/death registries. We used ICD‐10 codes suggesting the presence of liver cirrhosis, hepatocellular carcinoma, chronic liver failure, portal hypertension and oesophageal varices. OPCS‐4 codes referring to management of variceal bleeding, portal hypertension and ascites due to hepatic causes were also taken into account. The criteria are based on the selection of cases by Innes et al. [23] with minor modifications (Table S1). 80 patients who received a liver transplantation before their baseline assessment were excluded.

In both the AATD and PLHIV cohorts, LSMs were used as a surrogate for liver fibrosis and to classify patients according to established cut‐offs. Participants were categorised as having no/minimal fibrosis (LSM < 7.0 kPa), significant fibrosis (LSM ≥ 7.0 kPa), advanced fibrosis (LSM ≥ 10.0 kPa) or cirrhosis with potential portal hypertension (LSM > 15.0 kPa) based on previously validated thresholds [20, 21, 22, 24].

### 2.3 Plasma Proteomic Data

Plasma samples were collected from participants in all three cohorts and assessed using PEA technology. All proteomic data are reported as dimensionless normalised protein expression (NPX) values [12].

For the UKB cohort, samples from 54,219 participants who were part of the UKB Pharma Proteomics Project, including 46,595 randomly selected UKB participants from baseline assessments, 1268 participants belonging to the COVID‐19 repeat image study (with multiple visits) and 6376 participants directly selected by consortium members, were assessed with the Olink Explore platform, capturing 2923 unique proteins. After excluding 80 patients with previous liver transplantation, proteomic data from 52,998 participants were included in the analysis.

For the AATD cohort, plasma samples from 287 patients with Pi*ZZ genotype were analysed using the Olink Explore HT platform, which provided coverage of 5416 unique proteins.

The PLHIV cohort contributed plasma samples from 960 people that were analysed using the Olink Explore platform, capturing 2923 unique proteins, as described [16].

### 2.4 Statistical Analyses

All analyses were performed using the R environment (R Foundation, Vienna, Austria, version 4.4.1) and R Studio(version 2024.04.2+764) along with following R packages: dplyr [25], broom [26], ggplot2 [27], ggpubr [28], cowplot [29], limma [30], gtsummary [31], Hmisc [32], corrplot [33], SummarisedExperiment [34], glmnet [35], caret [36], pROC [37], precrec [38].

For proteomic analyses, differentially abundant proteins were identified using linear models with empirical Bayes moderation, with age and sex included as covariates. *p*‐values were adjusted for multiple testing using the false discovery rate (FDR) method. Proteins were considered significantly differentially abundant if their FDR was < 0.05 across a given condition. Proteins of interest were mapped to both publicly available tissue expression datasets (accessible at https://www.proteinatlas.org/humanproteome/tissue) and liver single‐cell RNA sequencing data (accessed via www.livercellatlas.org) [39].

For the comparison of protein levels between two groups, Wilcoxon rank sum tests were used. Box plots were used to visualise protein levels across different groups. Correlations between selected variables were assessed in a pairwise manner using Spearman's rank correlation test.

Demographic and clinical parameters were compared using linear regression models. When appropriate, models were adjusted for age, sex and BMI.

AST‐to‐platelet‐ratio index (APRI) and FIB4 were calculated using the following formulas: APRI = ((AST[IU/L]/34.8)/platelet count [×10^9^/L]); FIB4 = (age × AST[IU/L]/platelet count [×10^9^/L] × √ALT[IU/L]); and used with previously established cut‐offs for significant/advanced liver fibrosis  [40, 41, 42]. The LiverRisk score was calculated for the AATD and PLHIV cohorts as discussed previously using the web‐based calculator (https://www.liverriskscore.com) and was utilised with established cut‐offs [18]. As uploading individual‐level UKB data to external platforms is prohibited under UKB data governance policies and the formula for independent calculation is not publicly disclosed, the LiverRisk scores could not be calculated for UKB participants.

Associations between proteomic biomarkers and mortality outcomes were studied via logistic regression. For each protein of interest, a separate model was constructed to evaluate the relationship with both overall and liver‐related mortality (defined by ICD‐10 codes K70–K77 as the primary cause of death). All models were adjusted for age, sex and BMI as covariates. Results are presented as odds ratios (OR) with 95% confidence intervals per standard deviation increase in plasma levels. To ensure comparability across biomarkers with different expression ranges, protein levels were standardised prior to the analysis.

Data and results were reported using the TRIPOD reporting guidelines [43].

### 2.5 Development of Prognostic Score

To develop a prognostic score, we first assessed the ability of parameters to discriminate between patients developing a MALO and those without via logistic regression. Before any model development, the UKB participants with available PEA data were randomly divided into a training set (70%) and a test set (30%) using a stratified sampling approach to preserve a comparable distribution of MALO cases across subsets. All feature selection and model development was exclusively performed on the training set. All models included age and sex as covariates. For the modelling process, missing values were imputed using a modified k‐nearest neighbour approach, accounting for truncation at a minimum threshold (i.e., limit of detection) [44]. The subset of 20 proteomic variables, representing commonly altered proteins between the MALO‐associated signature in the UKB and fibrosis‐associated signatures in the AATD and PLHIV cohorts, were evaluated. Any feature reaching an area under receiver‐operating curve (AUROC) > 0.55 and *p* < 0.05 in a univariable analysis was used. All possible 4‐, 5‐ and 6‐protein combinations derived from these 20 features were systematically assessed, age and sex were added to all models as covariates. All models were analysed for collinearity, and variables with correlations > 0.7 or < −0.7 were not included in the same model to mitigate collinearity and increase the stability and accuracy of estimated coefficients. Models were considered if all coefficients presented a significant association (*p* < 0.05) with MALO, and their performance was assessed with AUROCs. For quality assessment, variable inflation factors were assessed for the selected model. Goodness of fit was assessed using the Hosmer‐Lemeshow test. Model calibration was further evaluated graphically using calibration plots, in which the mean predicted risk was plotted against the observed event rates across the deciles of predicted risks. The linearity between assessed variables and the logit of a future MALO was assessed both graphically and using the Box‐Tidwell test.

To enable a methodologically fair comparison between the developed PEA score and the clinical scores, comparator models using the individual components of APRI (AST and platelet count, with age and sex added as covariates) and FIB4 (AST, ALT, platelet count and age, with sex added as a covariate) were constructed. These comparator models were trained on the same 70% UKB training subset as the PEA score. The obtained model coefficients were then applied without refitting to the 30% UKB test subset and the UKB high‐risk subgroups.

### 2.6 External Validation of Developed Prognostic Score

The PEA score, APRI‐ and FIB4‐component models were validated externally in the AATD and PLHIV cohort. In both cohorts, the discriminative ability of the models was assessed using liver stiffness measurements (LSM) via FibroScan as the reference, with LSM ≥ 7.0 kPa defined as significant liver fibrosis. For the AATD cohort, analyses were performed both including and excluding patients with possible clinically significant portal hypertension (LSM > 15.0 kPa). All models were applied with coefficients fixed from the UKB training set derivation without refitting. The LiverRisk score was additionally included as a comparator in the external validation cohorts. Discriminative performances were assessed using AUROCs.

Given the significant class imbalance in the PLHIV cohort, model performances were additionally assessed using precision‐recall curves and negative predictive values at the Youden‐optimal threshold. Precision‐recall curves were generated using the *precrec R* package [38] and restricted to patients with complete available data for all compared indices.

To further assess the robustness of the PEA score in the PLHIV cohort, different sensitivity analyses were performed. First, the current class of antiretroviral therapy (ART) was included as additional covariates (non‐nucleoside reverse transcriptase inhibitors [NNRTI], integrase strand transfer inhibitors [INSTI] or protease inhibitors [PI]). Secondly, the cohort was restricted to participants diagnosed after 2007 and 2015, respectively, to reflect successive changes in standard‐of‐care ART initiation. Lastly, prior exposure to hepatotoxic nucleoside reverse transcriptase inhibitors (NRTI) was modelled both as a binary variable and as a cumulative duration.

## 3 Results

**FIGURE 1:**
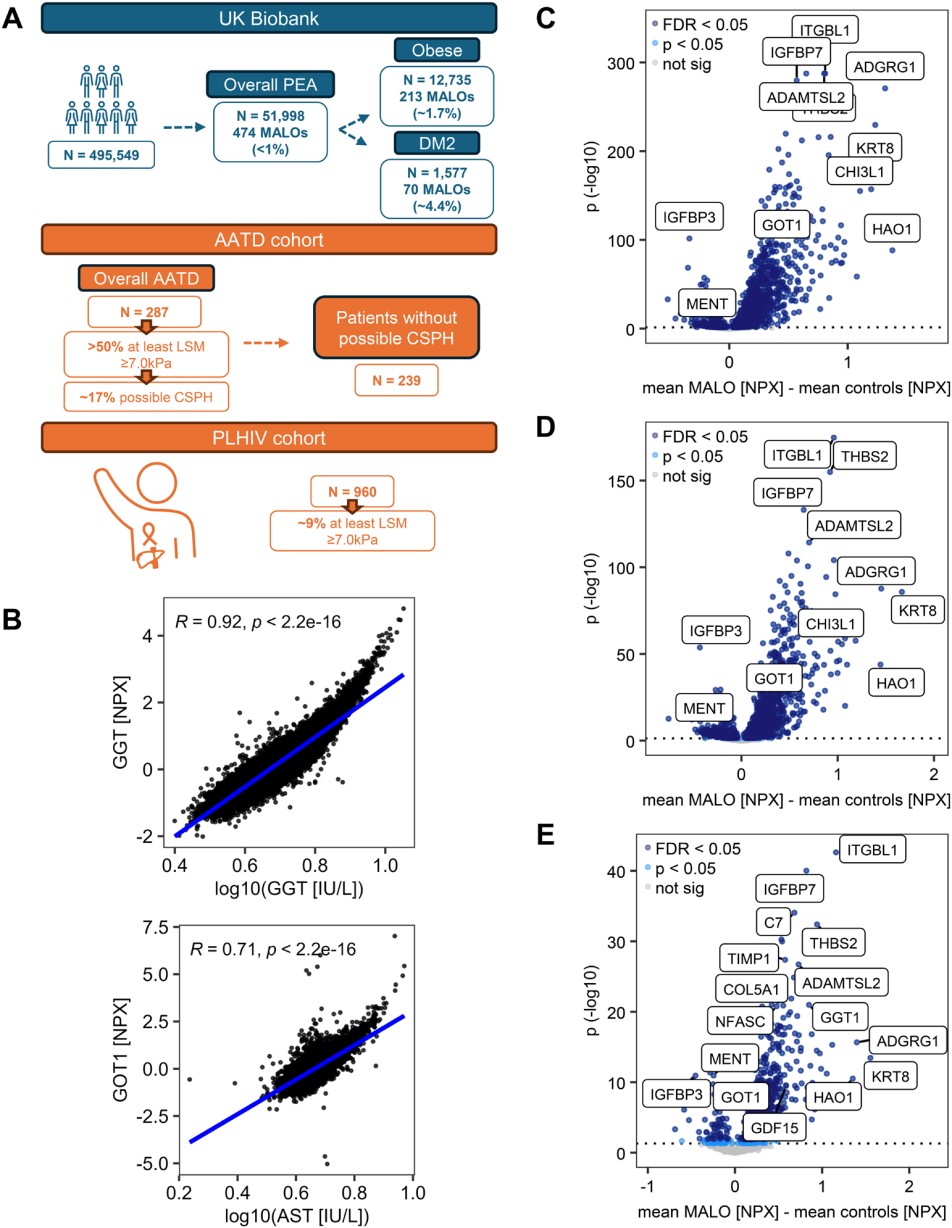
Proximity extension assay (PEA)‐based proteomics in participants from the UK Biobank cohort (UKB) with/without major adverse liver outcomes (MALOs). (A) Overview of the analysed cohorts. (B) Correlation between routine and PEA‐based measurements of gamma‐glutamyltransferase (PEA: GGT1; routine: GGT) and aspartate aminotransferase (PEA: GOT1, routine: AST). Spearman rank correlation coefficients and corresponding *p* values are shown. (C–E) Volcano plots of differentially abundant proteins between participants with/without future MALOs in the entire UKB cohort with available PEA analysis (C), in a subgroup of obese (BMI ≥ 30) participants (D) and a subgroup with type‐2 diabetes (DM2) (E). The dotted horizontal line reflects a *p* = 0.05. AATD, alpha1‐antitrypsin deficiency; possible CSPH, clinically significant portal hypertension, refers to LSM > 15.0 kPa; GOT1, glutamic‐oxaloacetic transaminase 1; LSM, liver stiffness measurement; PLHIV, people living with HIV.

Our study aimed to test the usefulness of a PEA proteomics to predict significant liver disease in three independent cohorts: (i) the community‐based UKB cohort; (ii) a multi‐centre AATD cohort; and (iii) a PLHIV cohort (Figure 1A). 51,998 participants from UKB with available routine lab and PEA measurement, representative of the entire UKB cohort, were assessed (Table 1). Two liver‐related biomarkers (GGT/GGT1 and AST/GOT1) were quantified by both routine methods and PEA, and their measurements displayed a strong correlation (*r* = 0.71 and *r* = 0.92, respectively, Figure 1B). Among the participants with available PEA data, 474 developed a MALO (Figure 1A; Table 1). Given that the occurrence of MALOs in this community‐based cohort was low (i.e., < 1%), we studied the performance of PEA proteomics in two higher‐risk subcohorts, that is, patients with baseline obesity (BMI ≥ 30) and DM2. The subcohorts encompassed 12,735 (obesity) and 1577 (DM2) participants who developed 213 (1.7%) and 70 (4.4%) MALOs, respectively (Figure 1A; Tables S2 and S3).

**TABLE 1:** Demographic and routine parameters of the UK Biobank (UKB) cohorts.

Characteristics	UKB cohort, *n* = 495,549	PEA cohort, *n* = 51,998	PEA cohort		*p* (A vs. B)
			Non‐MALOs (A), *n* = 51,524	MALOs (B), *n* = 474	
Age (years)	58 (50–63)	58 (50–64)	58 (50–64)	61 (56–65)	8.9E−17^a^
Sex (Female)	270,041 (54%)	28,104 (54%)	27,931 (54%)	173 (36%)	1.7E−14
BMI (kg/m^2^)	26.7 (24.1–29.9)	26.8 (24.2–29.9)	26.8 (24.2–29.9)	29.1 (25.7–33.1)	1.9E−28^a^
Type‐2 Diabetes	13,525 (2.7%)	1577 (3.0%)	1507 (2.9%)	70 (15%)	7.3E−28
Blood glucose (mmol/L)	4.93 (4.60–5.31)	4.93 (4.60–5.33)	4.93 (4.60–5.33)	5.18 (4.65–5.85)	3.3E−18^b^
HbA1C (mmol/mol)	35.2 (32.8–37.9)	35.4 (32.9–38.1)	35.3 (32.9–38.1)	36.9 (33.4–41.4)	1.3E−09^b^
Lipid‐lowering medication	78,004 (16%)	8745 (17%)	8607 (17%)	138 (29%)	2.4E−11
Total protein (g/L)	72.3 (69.7–75.1)	72.3 (69.7–75.1)	72.3 (69.7–75.0)	73.1 (70.0–76.9)	1.3E−07^b^
Albumin (g/L)	45.19 (43.48–46.92)	45.12 (43.40–46.88)	45.13 (43.40–46.88)	43.76 (41.77–45.94)	3.8E−25^b^
ALT (IU/L)	20 (15–27)	20 (15–27)	20 (15–27)	26 (18–45)	9.9E−69^b^
AST (IU/L)	24 (21–29)	24 (21–29)	**24 (21–29)**	**32 (24–47)**	**3.6E−224** ^b^
GGT (IU/L)	26 (19–41)	26 (19–41)	**26 (19–40)**	**66 (32–158)**	**< 1.9E−254** ^b^
ALP (IU/L)	80 (67–96)	81 (67–96)	81 (67–96)	94 (76–117)	2.3E−69^b^
Total bilirubin (μmol/L)	8.1 (6.4–10.4)	8.1 (6.4–10.4)	8.1 (6.4–10.4)	9.4 (7.0–12.7)	2.0E−20^b^
Direct bilirubin (μmol/L)	1.61 (1.30–2.09)	1.62 (1.30–2.11)	**1.62 (1.30–2.10)**	**2.09 (1.53–3.04)**	**2.1E−102** ^b^
Platelets (10^9^/L)	248 (213–287)	248 (213–287)	248 (213–287)	218 (173–263)	9.3E−22^b^

*Note:* The entire UKB cohort and its subset with available proteomic data (PEA cohort) are shown, the latter being subdivided into participants who did versus did not develop major adverse liver outcomes during the follow‐up (MALO/non‐MALO). Data are expressed as median (25th–75th percentile) for continuous variables and *n* (%) for categorical variables. *p‐*values for continuous variables were obtained from linear regression analyses. Associations between categorical variables were assessed using Fisher's exact test. Parameters with very low *p* values are shown as < 1.91E−254. Parameters with *p* < 1.00E−100 are highlighted in bold.

Abbreviations: ALP, alkaline phosphatase; ALT, alanine aminotransferase; AST, aspartate aminotransferase; GGT, gamma‐glutamyltransferase; HbA1C, haemoglobin A1c; PEA, proximity extension assay.

^a^ Without covariates.

^b^ With covariates age, sex and BMI.

In all assessed cohorts, participants developing MALOs were older, more often male and displayed higher levels of routinely assessed liver function tests (Tables 1, S2 and S3). After correction for multiple testing (FDR‐adjusted *p* value < 0.05), differential abundance analysis (age and sex as covariates) revealed 1748 differentially abundant proteins in the overall cohort and 1703 and 817 proteins in the obese and diabetic subcohorts (Figure 1C–E; Tables S4–S6).

### 3.1 Proteomic Signatures Associated With Advanced Liver Disease Across UKB Cohorts

**FIGURE 2:**
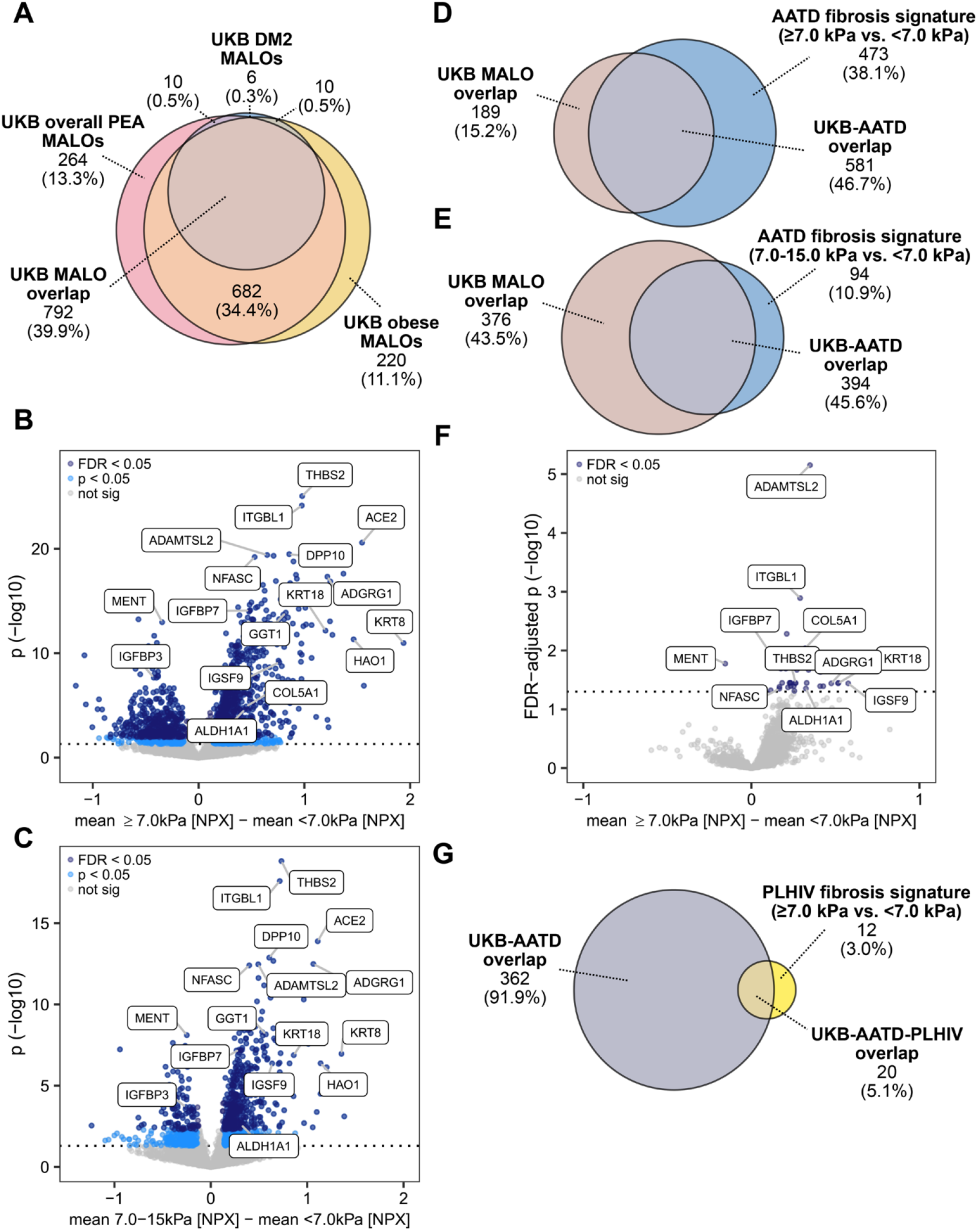
Identification of proteins consistently associated with significant liver disease across studied cohorts. (A) Venn diagram displays the overlap between signatures of proteins associated with MALO in three different subcohorts of the UKB (overall PEA cohort and subgroups of obese patients or patients with type‐2 diabetes [DM2]). 792 proteins were altered (false discovery rate (FDR) < 0.05) in all subcohorts. (B, C) Volcano plots displaying the results of the differential abundance analyses between patients with alpha1‐antitrypsin deficiency (AATD) with significant liver fibrosis (B: LSM ≥ 7.0 kPa, C: LSM between 7.0 and 15.0 kPa, that is, excluding patients with possible clinically significant portal hypertension) and those without (LSM < 7.0 kPa). 1266 and 548 proteins were differentially abundant (FDR < 0.05), respectively. (D, E) Overlaps between the proteomic signatures presented in B and C and the MALO‐associated features identified in the UKB shown as Venn diagrams. (F) Volcano plot visualises differentially abundant plasma proteins in people living with HIV (PLHIV) with high (≥ 7.0 kPa) versus low (< 7.0 kPa) LSM values as surrogates of significant liver fibrosis. 32 proteins were differentially abundant (FDR < 0.05). (G) Overlaps between proteomic signatures detected in the PLHIV cohort and the proteins that significantly differed in all previous analyses (UKB‐AATD overlap) are presented in a Venn diagram. 20 proteins were differentially abundant in all analyses. LSM, liver stiffness measurement; MALO, major adverse liver outcomes; PEA, proximity extension assay; UKB, the UK Biobank.

The overall pattern was similar in all assessed cohorts, with most proteins showing higher levels in participants with versus without future MALOs (Figure 1C–E). Only 24.2%, 31.6% and 7.3% of significantly altered proteins were diminished in the overall, obese and diabetic subcohorts with future MALOs (Figure 1C–E). 792 proteins were significantly altered in all analyses (Figure 2A). When the top 100 differentially abundant proteins were assigned to their tissue of origin, proteins produced by the liver, intestine or lymphoid tissues were the most common hits, while approximately 25% displayed low tissue specificity (Figure S1).

### 3.2 Validation of Proteomic Signatures in AATD and PLHIV Cohorts

To discover aetiology‐independent biomarkers, we turned to a cohort of 287 patients with severe AATD (Pi*ZZ genotype [14, 15]), whose liver fibrosis stage was characterised via LSM as well as clinically (Table 2). Although the baseline demographic parameters were comparable, subcohorts with higher fibrosis stages were more often male, had higher BMI, elevated liver enzymes and lower platelets (Table 2). Similar to UKB, PEA‐based GGT1/GOT1 strongly correlated with routine GGT/AST measurements (*r* = 0.90 and *r* = 0.86, Figure S2A,B). A comparison of patients with and without any clinically significant fibrosis (LSM ≥ 7.0 kPa vs. < 7.0 kPa) yielded 1266 significantly altered proteins (FDR < 0.05) (Figure 2B; Table S7). To study less advanced disease, we analysed the AATD subcohort without portal hypertension, that is, LSM ≤ 15.0 kPa. This assessment yielded 548 significantly altered (FDR < 0.05) proteins (Figure 2C; Table S8). The biomarkers identified in the AATD cohort strongly overlapped with the proteins found in the UKB analyses (i.e., 584 and 394 overlapping proteins (Figure 2D,E)). Unlike the AATD cohort, the PLHIV cohort only had a limited number of patients with significant fibrosis (LSM ≥ 7.0 kPa; *n* = 86; 9.0%; Table S9) and only 32 proteins differed significantly (FDR < 0.05) between the subgroups with high versus low LSM (Figure 2F). 20 of them, that is, the majority, were also significantly altered in all previous analyses (Figure 2G). Out of them, 19 were constantly increased and one was constantly decreased (MENT: C1orf56, Chromosome 1 open reading frame 56). Mapping these proteins to publicly available liver single‐cell sequencing data (Table 3) revealed that many are related to hepatic stellate cells (HSC) or epithelial cells (likely reflecting hepatocellular injury). Not surprisingly, the correlations between the biomarkers were weakest when the entire UKB cohort was considered and increased in cohorts enriched for participants with liver disease (Figures S3–S7). As expected, MENT displayed negative correlations while other biomarkers correlated positively (Figures S3–S7). Hepatocyte and HSC‐related biomarkers displayed stronger correlations with each other, while weaker correlations were seen between markers from different cellular sources. When assessing the association with overall/liver‐related death, all biomarkers but MENT presented with elevated OR. Although most of them were significantly associated with overall death, the association with liver‐related death was constantly stronger (Figures S8 and S9). To further validate the reliability of PEA quantification, we assessed THBS2 levels in the AATD cohort with a commercially available immunoassay kit. Both measurements displayed a strong correlation (*r* = 0.85, Figure S2C) and the PEA assessment yielded a somewhat higher AUROC for discriminating patients with versus without significant fibrosis (i.e., LSM ≥ 7.0 kPa vs. < 7.0 kPa; Figure S2D).

**TABLE 2:** Demographic and routine parameters of patients with severe alpha1‐antitrypsin deficiency (Pi*ZZ genotype).

Characteristics	*n*	AATD Pi*ZZ patients, LSM (kPa)					*p*
		< 7.0, *n* = 135	7.0–< 10, *n* = 55	10–< 15, *n* = 48	15–< 25, *n* = 19	≥ 25, *n* = 30	
Age (years)	285	56 (50–61)	58 (51–65)	59 (48–66)	61 (55–63)	60 (54–65)	0.71^a^
Sex (Female)	287	55 (41%)	11 (20%)	9 (19%)	2 (11%)	7 (23%)	0.002^a^
BMI (kg/m^2^)	285	24.8 (22.9–27.0)	27.3 (23.6–29.9)	26.5 (24.6–29.3)	25.6 (25.3–29.9)	26.7 (24.3–30.5)	0.80^a^
Diabetes (*n*)	283	3 (2.2%)	1 (1.9%)	5 (11%)	2 (11%)	5 (17%)	0.001^b^
ALT (IU/L)	284	31 (22–42)	37 (29–63)	43 (32–53)	53 (41–62)	57 (44–72)	8.18E−04^b^
AST (IU/L)	282	30 (23–36)	35 (30–50)	38 (32–45)	49 (38–69)	70 (49–96)	4.91E−09^b^
GGT (IU/L)	282	27 (21–42)	54 (34–89)	81 (42–129)	94 (53–264)	169 (87–301)	1.25E−05^b^
ALP (IU/L)	283	71 (60–85)	87 (65–104)	88 (72–102)	94 (74–139)	128 (104–189)	91.38E−05^b^
Total bilirubin (μmol/L)	280	8 (6–11)	11 (9–13)	11 (8–17)	13 (9–19)	22 (14–31)	2.37E−05^b^
Platelets (10^9^/L)	274	238 (196–278)	204 (166–242)	199 (134–242)	185 (123–248)	125 (101–150)	5.54E−07^b^

*Note:* Categorisation of patients is based on liver stiffness measurement (LSM), expressed in kPa. The subgroup ≥ 25 includes 19 patients with decompensated liver cirrhosis. Data are expressed as median (25th–75th percentile) for continuous variables and *n* (%) for categorical variables. *p‐*values for continuous variables were obtained from linear regression analyses. Associations between categorical variables were assessed using Fisher's exact test.

Abbreviations: AATD, alpha1‐antitrypsin deficiency; ALP, alkaline phosphatase; ALT, alanine aminotransferase; AST, aspartate aminotransferase; GGT, gamma‐glutamyltransferase; Pi*ZZ, homozygous Pi*Z mutation in alpha1‐antitrypsin gene.

^a^ Without covariates.

^b^ With covariates age, sex and BMI.

**TABLE 3:** Overview of proteins consistently associated with liver disease in all studied cohorts.

	MALO vs. non‐MALO (UKB)	MALO vs. non‐MALO (UKB obese)	MALO vs. non‐MALO (UKB DM2)	LSM ≥ 7.0 kPa vs. < 7.0 kPa (AATD)	LSM ≥ 7.0 kPa vs. < 7.0 kPa (PLHIV)	Liver cell subtype
THBS2	↑↑↑	↑↑↑	↑↑↑	↑↑↑	↑	Activated HSC
ITGBL1	↑↑↑	↑↑↑	↑↑↑	↑↑↑	↑	Activated HSC
ADAMTSL2	↑↑↑	↑↑↑	↑↑↑	↑↑↑	↑↑↑	Activated HSC
IGFBP7	↑↑↑	↑↑↑	↑↑↑	↑↑↑	↑	Activated HSC
ADGRG1	↑↑↑	↑↑↑	↑↑↑	↑↑↑	↑	NK cell
NFASC	↑↑↑	↑↑↑	↑↑↑	↑↑↑	↑	HSC
KRT18	↑↑↑	↑↑↑	↑↑↑	↑↑↑	↑	Epithelial
CD80	↑↑↑	↑↑↑	↑↑↑	↑↑↑	↑	Kupffer cell
CLSTN2	↑↑↑	↑↑↑	↑↑↑	↑↑↑	↑	Non‐specific
ENPP2	↑↑↑	↑↑↑	↑↑↑	↑↑	↑	Kupffer cell
CDH2	↑↑↑	↑↑↑	↑↑↑	↑↑↑	↑	Epithelial
ACY1	↑↑↑	↑↑↑	↑↑↑	↑	↑	Epithelial
ENG	↑↑↑	↑↑↑	↑↑↑	↑	↑	Endothelial
IGSF9	↑↑↑	↑↑↑	↑↑↑	↑↑↑	↑	T cell
ANGPT2	↑↑↑	↑↑↑	↑↑	↑	↑	Endothelial
DSC2	↑↑↑	↑↑↑	↑↑↑	↑↑↑	↑	Mesothelial
MENT	↓↓↓	↓↓↓	↓↓↓	↓↓↓	↓	NA
TFPI2	↑↑↑	↑↑↑	↑↑↑	↑↑↑	↑	Mesothelial
ALDH1A1	↑↑↑	↑↑↑	↑↑	↑	↑	Epithelial
CDH15	↑↑↑	↑↑↑	↑	↑	↑	Non‐specific

*Note:* ↑/↓ indicates proteins increased/decreased in diseased individuals. Significance levels are: ↑/↓ *p* < 0.05; ↑↑/↓↓ *p* < 0.001; ↑↑↑/↓↓↓ *p* < 0.0001. Proteins are mapped to publicly available liver single‐cell sequencing data [33].

Abbreviations: AATD, alpha1‐antitrypsin deficiency; ACY1, aminoacylase 1; ADAMTSL2, ADAMTS‐like protein 2; ADGRG1, adhesion G protein‐coupled receptor G1; ALDH1A1, aldehyde dehydrogenase 1A1; ANGPT2, angiopoietin 2; CD80, CD80 molecule; CDH2, cadherin 2; CDH15, cadherin 15; CLSTN2, calsyntenin 2; DM2, type‐2 diabetes; DSC2, desmocollin 2; ENG, endoglin; ENPP2, ectonucleotide pyrophosphatase/phosphodiesterase 2; HSC, hepatic stellate cell; IGFBP7, insulin‐like growth factor‐binding protein 7; IGSF9, Immunoglobulin superfamily member 9; ITGBL1, integrin beta‐like protein 1; KRT18, keratin‐18; LSM, liver stiffness measurement, expressed in kPa; MALO, major adverse liver outcomes; MENT, C1orf56 (chromosome 1 open reading frame 56); NA, not available; NFASC, neurofascin; NK, natural killer cell; PLHIV, people living with HIV; TFPI2, tissue factor pathway inhibitor 2; THBS2, thrombospondin‐2; UKB, the UK Biobank cohort.

### 3.3 Development and Validation of a Prognostic PEA Model and Its Components

We then took the 20 consistently altered biomarkers and performed a multivariable logistic regression to obtain a score reliably predicting future MALOs in the entire UKB cohort (termed PEA score; Tables 4 and S10; Figures S10 and S11). For this, UKB participants were randomly divided into a training (70%) and a test (30%) set using a stratified sampling approach. All feature selection and model development was performed exclusively on the training set. We systematically evaluated all possible four‐, five‐ and six‐protein combinations among the 20 candidate biomarkers and selected a 5‐feature signature, as this achieved an attractive predictive performance that was not substantially increased by the addition of further parameters. Alternative four‐, five‐ and six‐feature models showed comparable but less consistent performances across cohorts, further supporting the selection of our PEA score (Table S12).

**TABLE 4:** Composition of the proximity extension assay (PEA) score.

	Estimate	Standard error	*z* value	*p*	VIF
(Intercept)	−6.794	0.508	−13.359	< 2.20E−16	
ITGBL1	0.836	0.156	5.364	8.15E−08	3.952
ADAMTSL2	0.918	0.183	5.025	5.04E−07	3.80
IGFBP7	0.714	0.137	5.198	2.02E−07	2.108
KRT18	0.427	0.072	5.899	3.65E−09	3.033
ALDH1A1	−0.407	0.107	−3.792	0.0001	2.615
Age	0.021	0.008	2.537	0.011	1.064
Sex	0.389	0.091	4.257	2.07E−05	1.087

Abbreviations: ADAMTSL2, ADAMTS‐like protein 2; ALDH1A1, aldehyde dehydrogenase 1A1; IGFBP7, insulin‐like growth factor‐binding protein 7; ITGBL1, integrin beta‐like protein 1; KRT18, keratin‐18; VIF, variance inflation factor.

To assess the added value of the PEA score beyond established clinical tools, we compared its performance against APRI and FIB4, two widely used indices for liver fibrosis [42, 45]. In addition, to enable a methodologically fair comparison, we constructed comparator models incorporating their individual components, which were trained on the same 70% training set and applied to the 30% test without refitting.

**FIGURE 3:**
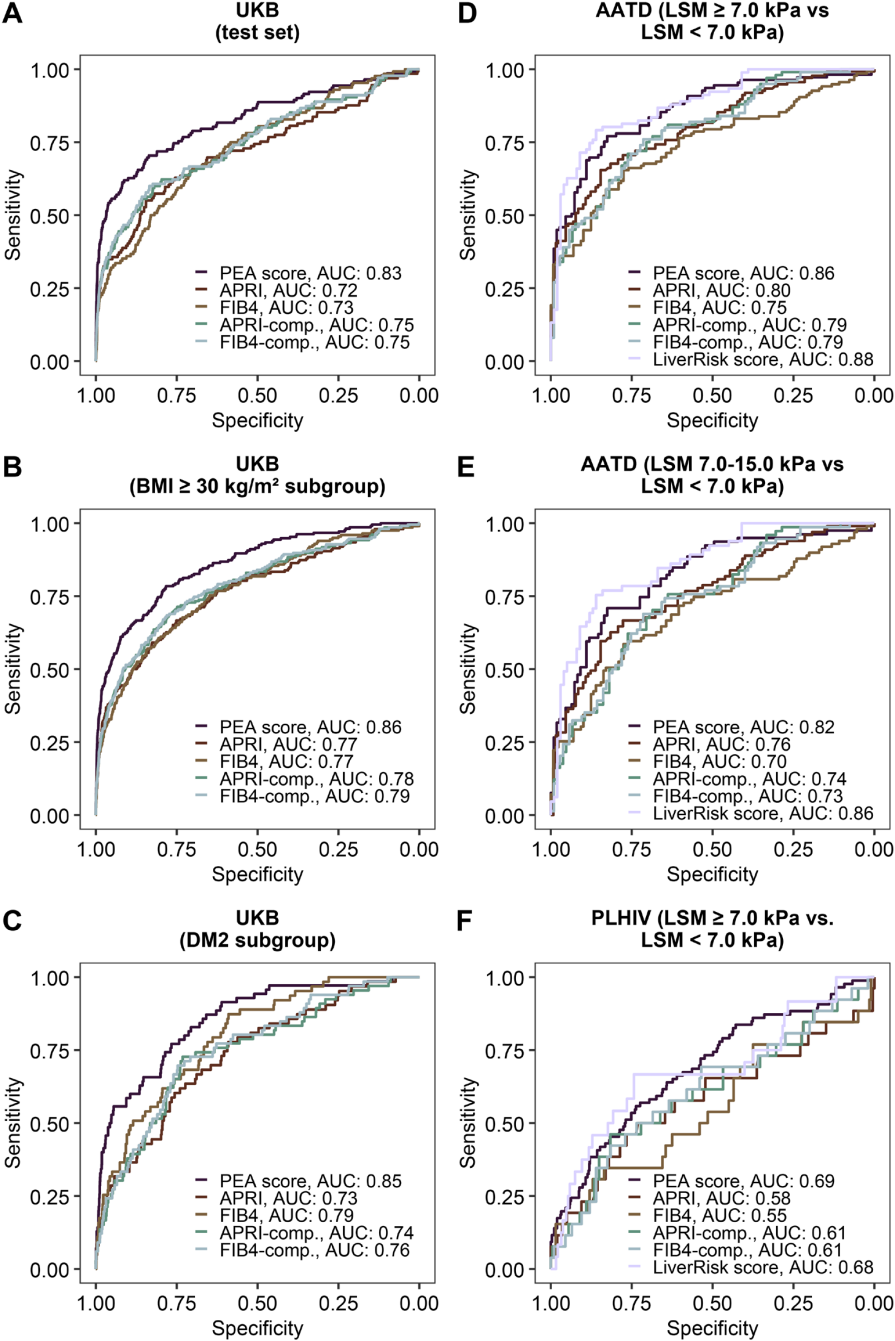
Comparison of a proximity extension assay (PEA) score with established liver fibrosis indices across patient cohorts. Receiver‐operating curves (ROCs) displaying the performance of a PEA score (comprising ITGBL1, ADAMTSL2, IGFBPB7, KRT18, ALDH1A1, age and sex added as covariates) in comparison to AST‐to‐platelet‐ratio index (APRI), Fibrosis‐4 index (FIB4), APRI‐ and FIB4‐component models and the LiverRisk score. The PEA score and the APRI‐ and FIB4‐component models (which were trained on the individual components of APRI [AST, platelet count, with age and sex added as covariates] and FIB4 [AST, ALT, platelet count, age, with sex added as covariate]) were developed on the UKB training set and then applied with fixed coefficients to all cohorts including the UKB test subcohort. APRI and FIB4 indicate the original scores that were applied without refitting. (A–C) Prediction of major adverse liver outcomes (MALOs) in the UK Biobank (UKB) test set with available PEA‐data [A], and subgroups of UKB patients with obesity (BMI ≥ 30 kg/m^2^) [B] or type‐2 diabetes (DM2) [C]. (D, E) Differentiation between patients with alpha1‐antitrypsin deficiency (AATD) based on liver stiffness measurements (LSM) via FibroScan. Patients with significant liver fibrosis (D: LSM ≥ 7.0 kPa, E: LSM between 7.0 and 15.0 kPa, that is, excluding patients with possible clinically significant portal hypertension) were compared against those without (LSM < 7.0 kPa). (F) LSM‐based differentiation between people living with HIV (PLHIV) with (LSM ≥ 7.0 kPa) and without significant liver fibrosis (LSM < 7.0 kPa). The areas under the receiver‐operating curve (AUROCs) are shown. The LiverRisk score could not be calculated for UKB participants due to data protection restrictions (panels A–C).

The PEA score predicted MALOs with an AUROC of 0.84 in the UKB training set and 0.83 in the test set, outperforming APRI (AUROC = 0.72) and FIB4 (AUROC = 0.73), as well as their respective comparator models (APRI‐comp, AUROC = 0.75; FIB4‐comp, AUROC = 0.75) (Figure 3A) and the same was true in the obese/diabetic UKB subcohorts (Figure 3B,C). Calibration plots (Figure S12) demonstrated good agreement between predicted and observed event rates for the PEA score, as well as the APRI‐ and FIB4‐comparator models, whereas APRI and FIB4 showed slightly stronger deviations from the calibration line.

In the AATD and PLHIV validation cohorts, the PEA score maintained its superior performance compared to APRI/FIB4, as well as their comparator models and achieved comparable performance to the LiverRisk score (Figure 3D–F). Given the significant class imbalance in the PLHIV cohort (86 out of 960 patients with LSM ≥ 7.0 kPa), we additionally assessed the model performances using precision‐recall analysis and evaluated negative predictive values (NPVs) at clinically relevant thresholds. The precision‐recall analysis confirmed the superiority of the PEA score (AUPRC = 0.355) over APRI (AUPRC = 0.241) and FIB4 (AUPRC = 0.208) and their corresponding comparator models (AUPRCs 0.219 and 0.21, respectively) in the subset with complete available data (*N* = 226, Figure S13). At the Youden‐optimal threshold, the PEA score achieved the highest NPV (0.959) and sensitivity (0.846), suggesting its utility for ruling out clinically relevant liver fibrosis in this population (Table S13). To assess the robustness of these findings to potential confounding through antiretroviral therapy (ART) regimen, sensitivity analyses adjusting for currently used classes of ART, prior exposure to nucleoside reverse transcriptase inhibitors (NRTIs) or the treatment era were performed. Protein coefficients remained directionally consistent across all analyses (Figure S14, Table S14).

**FIGURE 4:**
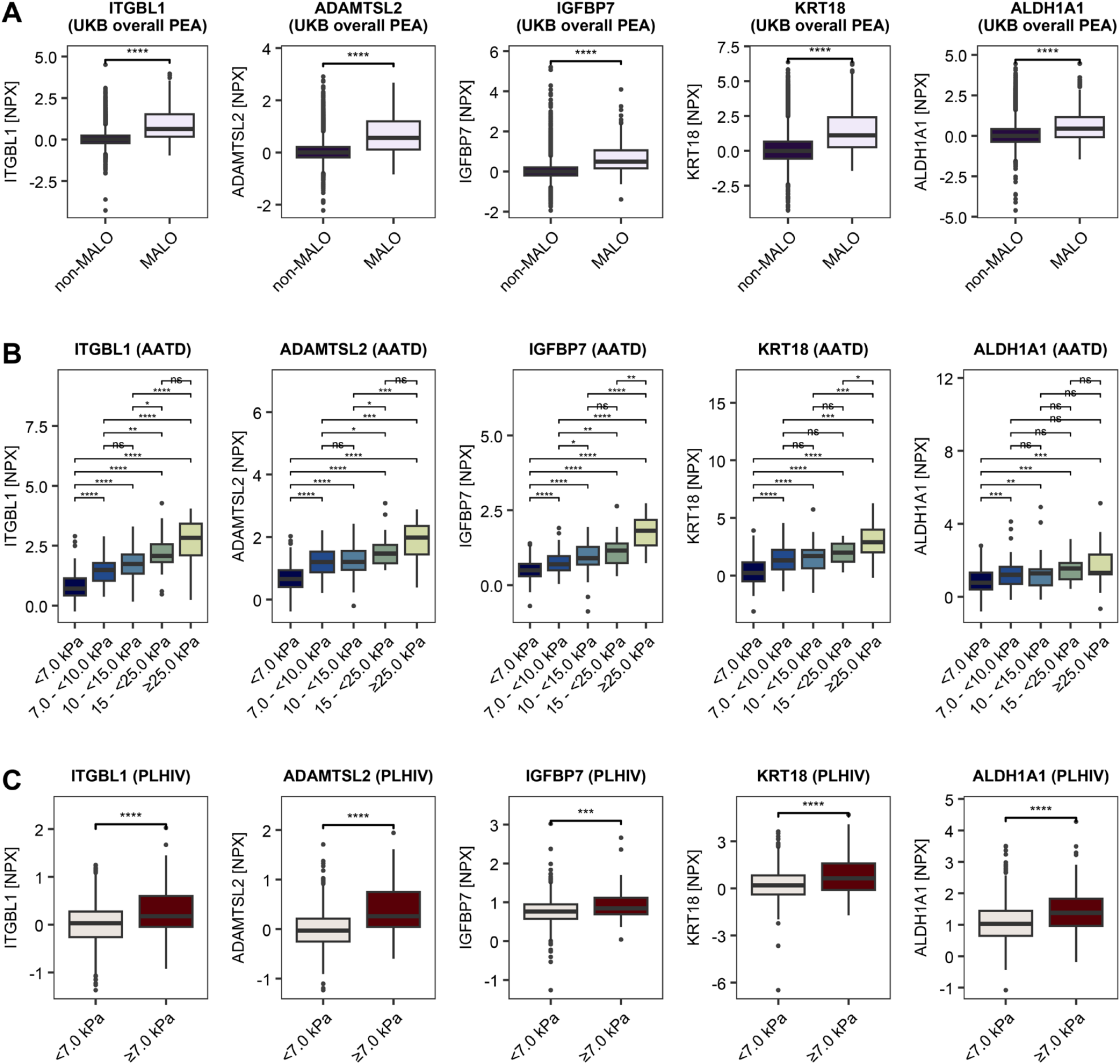
Analysis of the identified proteomic markers associated with significant liver disease across different patient populations. (A) Plasma levels (normalised protein expression [NPX] values) of five protein markers (ITGBL1, ADAMTSL2, IGFBP7, KRT18 and ALDH1A1) in the overall UK Biobank (UKB) population with available proximity extension assay (PEA)‐based proteomic data. Baseline measurements from patients with (MALO) and without major adverse liver outcomes during follow‐up (non‐MALO) are compared. (B) Plasma levels of the above‐described biomarkers in patients with alpha1‐antitrypsin deficiency (AATD) with different liver fibrosis stages as indicated by liver stiffness measurement (LSM) (< 7.0, 7.0–10.0, 10.0–15.0, 15.0–25.0 and ≥ 25.0 kPa). (C) Plasma levels of the above‐described biomarkers in people living with HIV (PLHIV) with versus without LSM‐based significant fibrosis (i.e., LSM ≥ 7.0 vs. < 7.0 kPa). Box plots indicate the median (central line), 25th–75th percentiles (box) and smallest/largest non‐outlier values (whiskers); outliers are depicted as black dots. Significance levels are indicated as follows: *p < 0.05, **p < 0.01, ***p < 0.001, ****p < 0.0001 (Wilcoxon rank‐sum test). ADAMTSL2, ADAMTS‐like protein 2; ALDH1A1, aldehyde dehydrogenase 1A1; IGFBP7, insulin‐like growth factor‐binding protein 7; ITGBL1, integrin beta‐like protein 1; KRT18, keratin‐18.

An assessment of the five components of the PEA score; integrin beta‐like protein 1 (ITGBL1), ADAMTS‐like protein 2 (ADAMTSL2), insulin‐like growth factor‐binding protein 7 (IGFBP7), keratin‐18 (KRT18) and aldehyde dehydrogenase 1A1 (ALDH1A1), revealed stronger correlations between the HSC‐related (i.e., ITGBL1, ADAMTSL2 and IGFBP7) and epithelial markers (i.e., ALDH1A1 and KRT18) but somewhat less pronounced associations between the groups (Figures S3–S7). Notably, the levels of all biomarkers significantly differed among participants with/without future MALOs as well as with lower versus higher liver fibrosis/disease surrogates APRI, FIB4, LSM and the LiverRisk score (Figures 4, S15–S17).

Collectively, our data indicate that PEA reliably quantifies several liver‐related parameters and that PEA‐based assessment can be used to develop prognostically useful liver scores.

## 4 Discussion

The study objective was to evaluate the usefulness of the emerging PEA technique as a source of liver‐related biomarkers. First, we demonstrated that analyses of three different cohorts yielded similar top biomarkers even though they were performed in different PEA facilities, which confirms the robustness and standardisation of the assay. In line with that, the levels of several parameters measured by PEA strongly correlated with values obtained by alternative techniques. However, this may not be the case for all biomarkers [39] and because of that, the markers of interest need to be individually validated. Second, we identified 20 biomarkers that were consistently altered in all assessed cohorts and used a bioinformatic analysis to obtain a novel five‐feature score. This comprised three HSC‐related and two epithelial proteins. Notably, the HSC‐based markers were previously reported as attractive liver fibrosis surrogates [6, 10, 46, 47, 48, 49, 50]. In addition, KRT18 is frequently assessed by the well‐established M30/M65‐immunoassays and is considered a marker of histological disease severity [46, 47, 48]. ALDH1A1, as the second epithelial protein, is strongly expressed in pericentral hepatocytes and might therefore be particularly useful to detect an injury in this compartment [6, 49, 50, 51]. The fact that these five biomarkers emerged from an unbiased analysis of ~3000 proteins strongly supports the importance of HSC and epithelial surrogates as prognosticators of liver disease severity. This is in line with data from a different affinity‐based proteomic technique as well as mass spectrometry‐based analysis, which identified similar proteomic signatures [6, 10, 52, 53]. Notably, the 20 consistently altered proteins also contained several biomarkers linked to inflammation and endothelial function. The presence of an endothelial‐related signature is not surprising, as endothelial dysfunction is important for liver disease progression and the development of portal hypertension, but might be less relevant in earlier disease stages [54, 55]. Similarly, the identification of Kupffer cell‐related markers is well in line with their importance in both liver injury and homeostasis [56]. Further studies are needed to test whether the addition of these representatives provides further prognostic benefits.

Our analysis has several important strengths. First, all assessed cohorts included a systematic evaluation of key liver‐related parameters (i.e., liver fibrosis or MALO) that are strongly related to liver‐related death [23]. Second, while metabolic liver disease is the prevailing liver disease aetiology in the UKB [57], the results were validated in a cohort with a genetically determined, proteotoxic liver injury [15] as well as an HIV‐related cohort [16]. To the best of our knowledge, this is the first large‐scale serum proteomics study addressing AATD‐associated liver disease, which is a field of growing interest given the ongoing development of targeted therapies [58] and our findings may support these efforts. The strong overlap between biomarkers seen in the UKB and the AATD cohorts suggests that the detected signature reflects core fibrosis‐related mechanisms rather than aetiology‐specific patterns, which meshes well with their ability to reflect essential fibrosis‐related processes such as HSC activation. Another strength is the longitudinal design of the UKB analysis; we were able to prognosticate the development of MALOs, an outcome that is challenging to study due to its relatively low incidence and slow liver fibrosis progression [59, 60]. At the same time, we evaluated the performance of the derived score in cohorts characterised by cross‐sectional LSM, including the PLHIV cohort, which predominantly comprised individuals with no or minimal fibrosis, as well as three clinically‐relevant high‐risk populations (i.e., diabetes, obesity and AATD).

Notably, the described score outperformed the widely‐used APRI/FIB4 scores and was comparable to or slightly better than the recently described LiverRisk score. However, its discriminative ability was lower in the PLHIV cohort. This finding most likely reflects the specific composition of this cohort, in which the majority of patients had minimal‐to‐moderate fibrosis and the LSM that was used as a comparator has a suboptimal discriminative ability in this range [61]. This suggests that the PEA score may be particularly well‐suited for identifying advanced fibrosis and predicting clinically relevant outcomes, whereas its utility in detecting early‐stage significant fibrosis requires further evaluation in appropriate cohorts comprising the full fibrosis spectrum.

Importantly, the UKB allowed a true prognostic modelling using longitudinal MALO data, whereas the validation in the AATD and PLHIV cohorts relied on cross‐sectional LSM as diagnostic surrogate. While the fibrosis stage is strongly associated with future liver‐related events, this approach does not fully validate the score's ability to predict longitudinal outcomes. This limitation reflects the current lack of large, prospective cohorts with available longitudinal outcome data and should be addressed in future studies.

Compared to other proteomic approaches, our PEA score achieved performance comparable to or slightly higher than scores described for the SomaScan technique, despite using fewer biomarkers [62]. However, head‐to‐head studies are needed to compare both methods.

Despite the above‐described strengths, our study has several limitations. Most participants in the assessed cohorts were of European descent [20, 63], and validation in ethnically diverse populations will be necessary before broad clinical implementation can be considered. Furthermore, additional longitudinal cohorts with sufficient cases of MALOs would be desirable to confirm the prognostic performance across different disease aetiologies. Our analyses also indicate that the score performs less well in patients with minimal‐to‐moderate liver disease, underlining the need for further evaluation across the entire spectrum of fibrosis stages. Regarding the latter, UKB cohort offers only a basic liver fibrosis assessment and our study therefore likely included at least some subjects with clinically apparent liver cirrhosis. In addition, our analyses focused primarily on hepatocellular liver disorders and the applicability of our score to cholestatic diseases remains to be tested.

Our work should be considered a proof‐of‐concept study demonstrating the feasibility and biological relevance of large‐scale PEA‐based liver biomarker discovery. Further optimisation, simplification and development of smaller, well‐validated, cost‐efficient liver panels will be required before a potential implementation in the clinical routine. In conclusion, our study demonstrates the usefulness of a new PEA proteomic technique in determining the liver fibrosis stage and in prognosticating the development of MALOs. Given that this non‐invasive approach requires only a small sample volume, is standardised across facilities and quantifies proteins across an extensive dynamic range of protein concentrations [7], it may become a valuable tool for both patient stratification and drug development.

## Author Contributions

Study concept and design: K.R., P.S. Acquisition of data: S.B., K.R., A.S.K, C.S., M.F., L.E.v.E., L.A.B.J., T.O. Analysis and interpretation of data: S.B., A.S.K., K.R., P.T. Drafting of the manuscript: S.B., K.R., P.S. Critical revision of the manuscript for important intellectual content: K.R., P.S. Figures and tables: S.B., K.R., J.A.B. Statistical analysis: S.B., A.S.K., K.R., P.T. Obtained funding: P.S., C.K., B.Z., M.L., P.K. Administrative, technical or material support: C.S., M.F., C.K., B.Z., M.L., P.K., L.E.v.E., L.A.B.J., T.O. Study supervision: K.R., P.S. All authors had full access to all the data and approved the final version of this manuscript. All authors take responsibility for the integrity of the data and the accuracy of the data analysis.

## Funding

This work was supported by the EASL registry grant on alpha1‐antitrypsin‐related liver disease, the DFG grants STR1095/6‐1, SFB 1382 (ID 403224013), unrestricted research grants from CSL Behring and Arrowhead Pharmaceuticals and the German Liver Foundation.

## Ethics Statement

For analysis involving the UK Biobank, all data handling was performed in accordance with the guidelines and regulations approved by the UK Biobank (application number 148742). The Ethics Committee of Aachen University (Aachen: EK 173/15) provided ethical approval for the Alpha 1 antitrypsin cohort. All participants were assessed following the ethical principles of the Helsinki Declaration (Hong Kong amendment) and Good Clinical Practice (European guidelines).

## Consent

All participants gave written informed consent.

## Conflicts of Interest

P.S. reports receiving grants and honoraria from Sanofi, Arrowhead Pharmaceuticals, CSL Behring, Grifols Inc., consulting fees or honoraria from Alnylam Pharmaceuticals, AiRNA, Arrowhead Pharmaceuticals, Beam Pharmaceuticals, BioMarin Pharmaceutical, Dicerna Pharmaceuticals, GondolaBio, GSK, IPSEN Pharmaceuticals, Intellia Pharmaceuticals, Korro Bio, Takeda Pharmaceuticals, Tessera Therapeutics, Novo Nordisk and Ono Pharmaceuticals, participating in leadership or fiduciary roles in Alpha1‐Deutschland, Alpha1 Global and material transfer support for Vertex Pharmaceuticals and Dicerna Pharmaceuticals. M.F. received consulting fees from Takeda Pharmaceuticals and honoraria from CSL Behring, Grifols Inc. and Takeda Pharmaceuticals. S.B., A.S.K., J.A.B., C.S., L.E.v.E., L.A.B.J., T.O., P.T. and K.R. report no conflicts of interest. C.K., B.Z., M.L. and P.K. are employees of Sanofi.

## Supporting information


**Figure S1:** apt70656‐sup‐0001‐supinfo.docx.

**Table S1:** apt70656‐sup‐0002‐Tables.xlsx.

## Data Availability

Data are available upon reasonable request to the corresponding author.
