# Supplementary material for: Affinity Proteomics‐Based Non‐Invasive Detection of Clinically Significant Liver Disease
Source: Aliment Pharmacol Ther. 2026 Apr 16;64(1):74–88. doi: 10.1111/apt.70656 (PMC13251591; doi:10.1111/apt.70656)
Supplement: Supplementary file 1 — Figure S1: apt70656‐sup‐0001‐supinfo.docx. [file APT-64-74-s002.docx]

**Affinity proteomics-based non-invasive detection of clinically significant liver disease**

Sriram Balasubramani^1,*^, Katharina Remih^1,*^, Anna Sophie Karl^1^, Julia Alexandra Borchert^1^, Christina Schrader^1^, Malin Fromme^1^, Can Kayatekin^2^, Bailin Zhang^2^, Mikhail Levit^2^, Pavithra Krishnaswami^2^, Louise E. van Eekeren^3^, Leo A. B. Joosten^3,4^, Twan Otten^3^, Petra Tomanová^5^, Pavel Strnad^1,§^

^1^ Medical Clinic III, Gastroenterology, Metabolic diseases and Intensive Care, University Hospital RWTH Aachen, Health Care Provider of the European Reference Network on Rare Liver Disorders (ERN RARE LIVER), Aachen, Germany.

^2^ Sanofi, Cambridge, Massachusetts, United States of America

^3^ Department of Internal Medicine, Radboud University Medical Centre, Nijmegen, the Netherlands

^4^ Department of Medical Genetics, Iuliu Hatieganu University of Medicine and Pharmacy, Cluj-Napoca, Romania

^5^ Prague University of Economics and Business, Prague, Czech Republic

*shared first authors; § corresponding author

Table of Contents

[Supplementary Figures 2](#_Toc210829391)

[Supplementary Tables 22](#_Toc210829392)

# Supplementary Figures


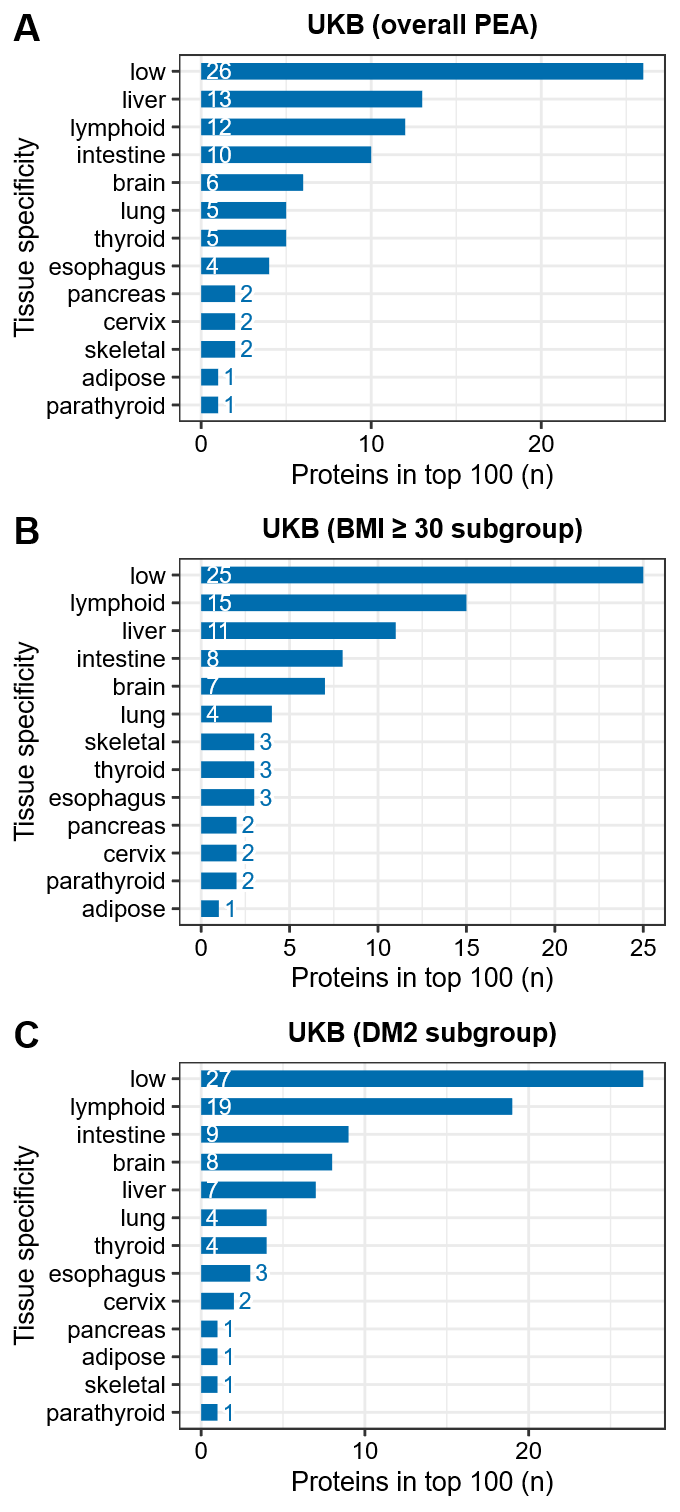


**Fig. S1. Mapping of the top 100 differentially abundant proteins in the** **UK Biobank (UKB) cohort to their tissue of origin.** Proteins that discriminate between subjects with/without future major adverse liver outcomes (MALOs) were assessed. The plots show the absolute number of proteins mapped to a specific tissue in (A) the entire proteomic cohort, (B) a subgroup of obese (BMI ≥30) patients and (C) a subgroup with type-2 diabetes (DM2). A single protein may be specific to more than one tissue. 20 (in A/C) and 23 (in B) proteins could not be assigned and are not shown in the plots above .


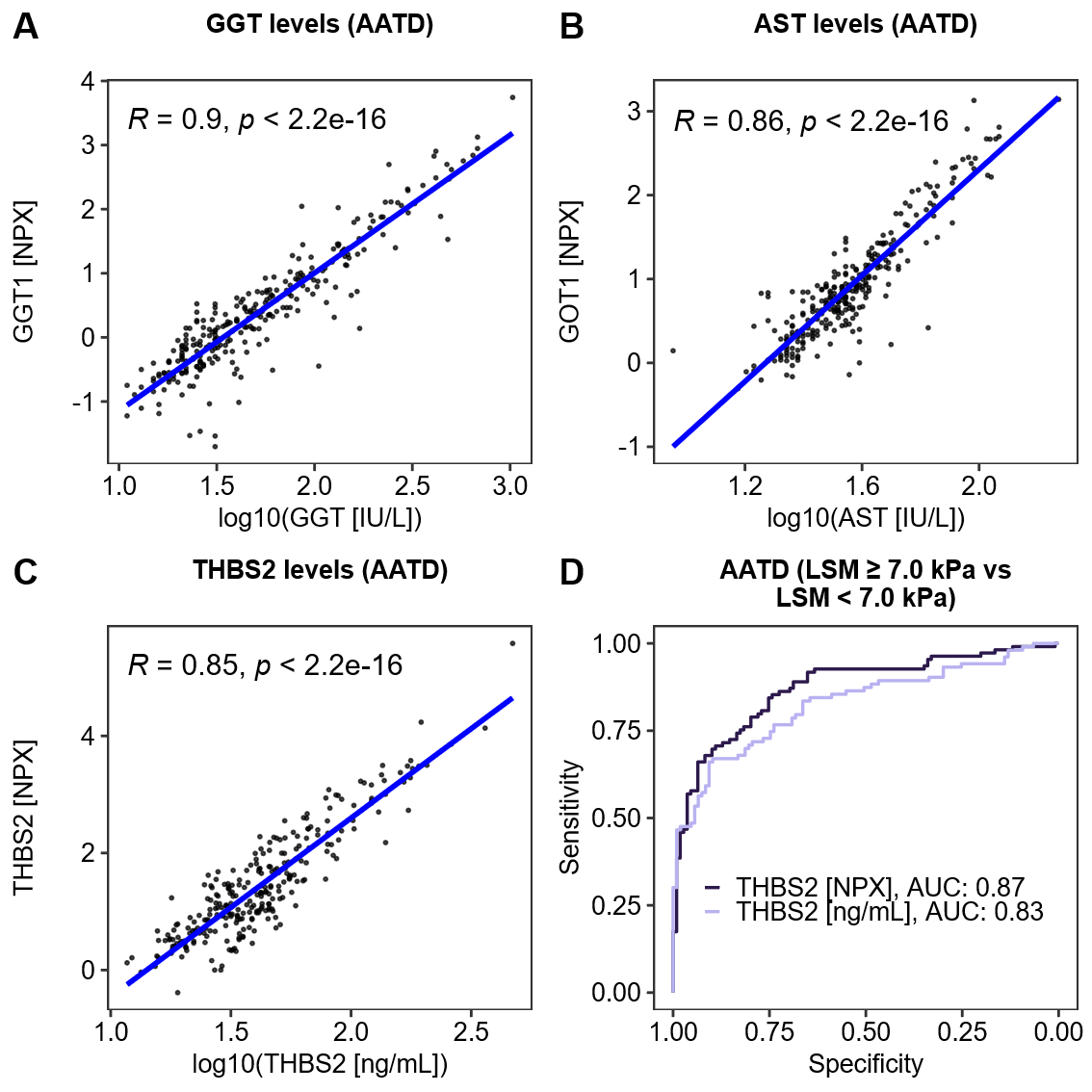


**Fig. S2: Comparing serum and proteomic measurements of biomarkers in the cohort of severe alpha1-antitrypsin deficiency (AATD) subjects.** A/B: Scatter plot depicts correlation of routine log10-transformed (in IU/L) and corresponding proximity extension assay (PEA)-based measurements of gamma-glutamyltransferase (GGT/GGT1) and aspartate aminotransferase (AST/GOT1) serum levels. C: Depicts the correlation between log10-transformed serum concentrations of thrombospondin-2 (THBS2, in ng/mL) determined via immunoassay (x-axis) and corresponding PEA measurements (Olink® platform, y-axis, in normalised protein expression [NPX] values). Blue lines represent the linear regression fit. Each dot represents an individual sample. Spearman rank correlation factors and *P* values are depicted. D: Receiver operating curves (ROCs) display the ability of a PEA- and immunoassay-based THBS2 levels (in ng/mL) to distinguish AATD subjects with vs. without significant liver fibrosis assessed through liver stiffness measurements (LSM) via FibroScan®. AATD subjects with LSM ≥7·0 kPa and LSM <7·0 kPa were compared. The area under the receiver operating curve (AUROC) is shown.


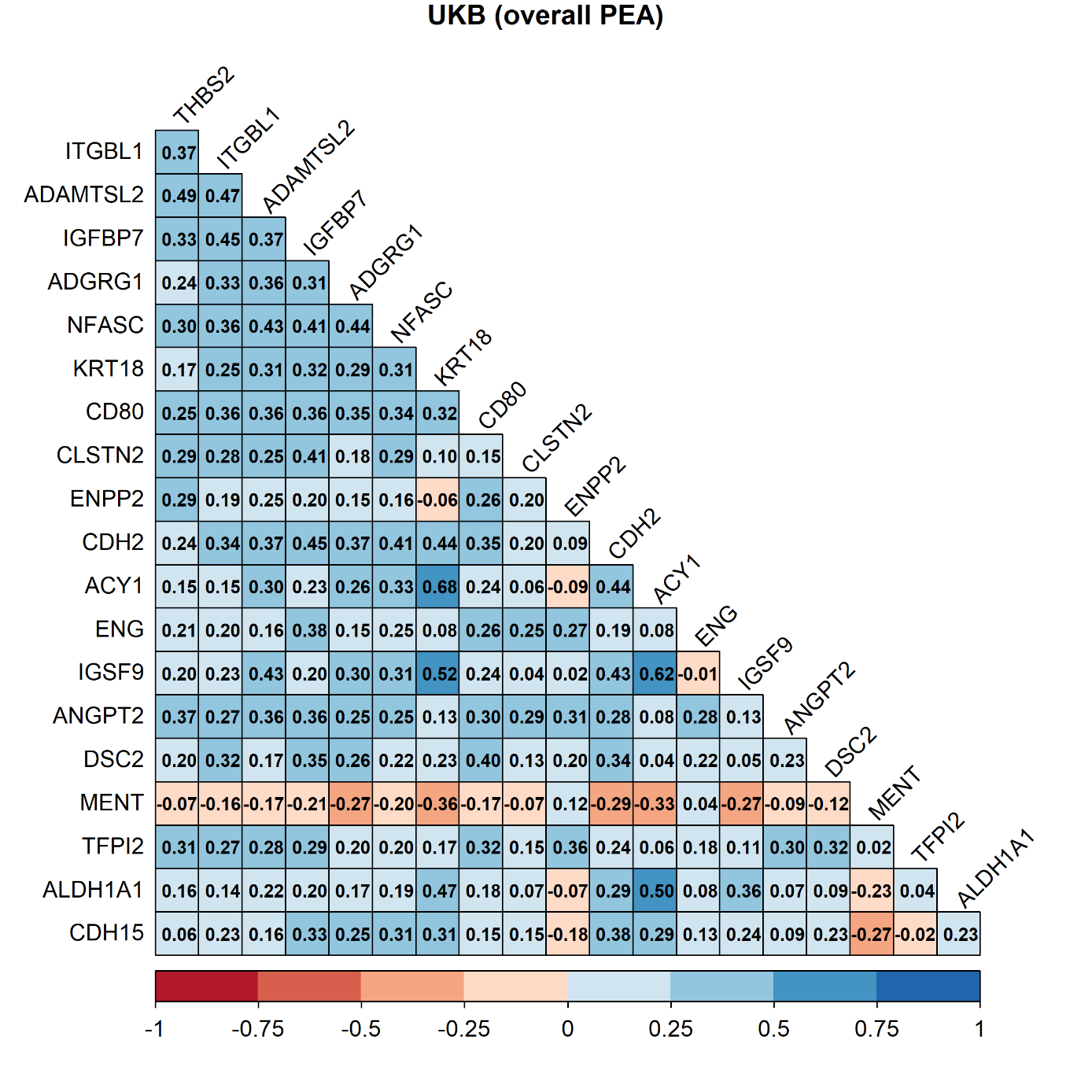


**Fig. S3: Correlation of 20 biomarkers in the UK Biobank (UKB) cohort with available proximity extension assay (PEA) proteomic data.** Displayed are the Spearman rank correlation coefficients. *ACY1: aminoacylase 1; ADAMTSL2: ADAMTS-like protein 2; ADGRG1: adhesion G protein-coupled receptor G1; ALDH1A1: aldehyde dehydrogenase 1A1; ANGPT2: angiopoietin 2; CD80: CD80 molecule; CDH2: cadherin 2; CDH15: cadherin 15; CLSTN2: calsyntenin 2; DSC2: desmocollin 2; ENG: endoglin; ENPP2: ectonucleotide pyrophosphatase/phosphodiesterase 2; IGFBP7: insulin-like growth factor-binding protein 7; IGSF9: Immunoglobulin superfamily member 9; ITGBL1: integrin beta-like protein 1; KRT18: keratin-18; MENT: C1orf56 (chromosome 1 open reading frame 56); NFASC: neurofascin; TFPI2: tissue factor pathway inhibitor 2; THBS2: thrombospondin-2.*


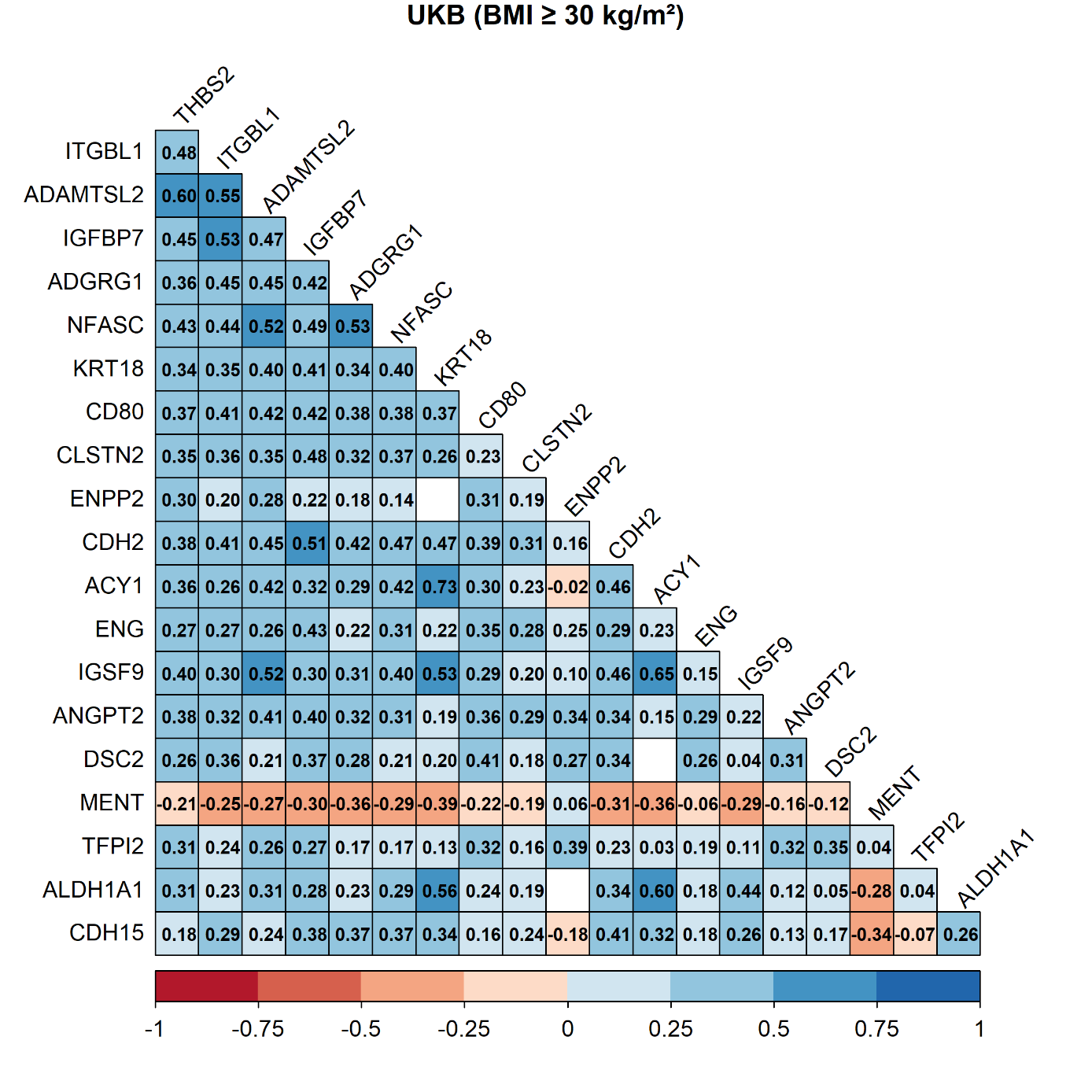


**Fig. S4: Correlation of 20 biomarkers in a subgroup of obese (BMI ≥30 kg/m²) UK Biobank (UKB) participants.** Displayed are the Spearman rank correlation coefficients. *ACY1: aminoacylase 1; ADAMTSL2: ADAMTS-like protein 2; ADGRG1: adhesion G protein-coupled receptor G1; ALDH1A1: aldehyde dehydrogenase 1A1; ANGPT2: angiopoietin 2; CD80: CD80 molecule; CDH2: cadherin 2; CDH15: cadherin 15; CLSTN2: calsyntenin 2; DSC2: desmocollin 2; ENG: endoglin; ENPP2: ectonucleotide pyrophosphatase/phosphodiesterase 2; IGFBP7: insulin-like growth factor-binding protein 7; IGSF9: Immunoglobulin superfamily member 9; ITGBL1: integrin beta-like protein 1; KRT18: keratin-18; NFASC: neurofascin; MENT: C1orf56 (chromosome 1 open reading frame 56); TFPI2: tissue factor pathway inhibitor 2; THBS2: thrombospondin-2.*


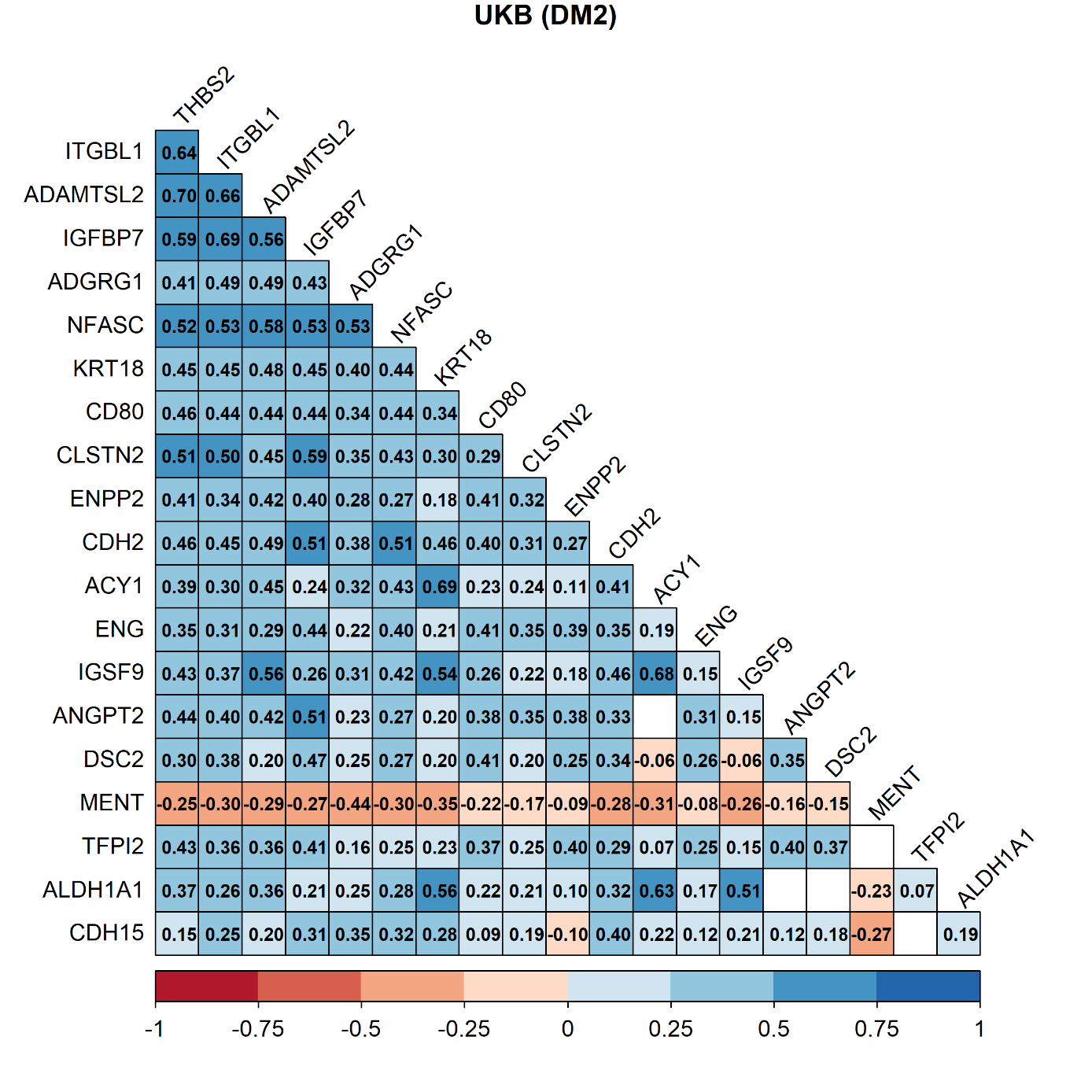


**Fig. S5: Correlation of 20 biomarkers in a subgroup of type-2 diabetic (DM2) UK Biobank (UKB) participants.** Displayed are the Spearman rank correlation coefficients. *ACY1: aminoacylase 1; ADAMTSL2: ADAMTS-like protein 2; ADGRG1: adhesion G protein-coupled receptor G1; ALDH1A1: aldehyde dehydrogenase 1A1; ANGPT2: angiopoietin 2; CD80: CD80 molecule; CDH2: cadherin 2; CDH15: cadherin 15; CLSTN2: calsyntenin 2; DSC2: desmocollin 2; ENG: endoglin; ENPP2: ectonucleotide pyrophosphatase/phosphodiesterase 2; IGFBP7: insulin-like growth factor-binding protein 7; IGSF9: Immunoglobulin superfamily member 9; ITGBL1: integrin beta-like protein 1; KRT18: keratin-18; MENT: C1orf56 (chromosome 1 open reading frame 56); NFASC: neurofascin; TFPI2: tissue factor pathway inhibitor 2; THBS2: thrombospondin-2.*


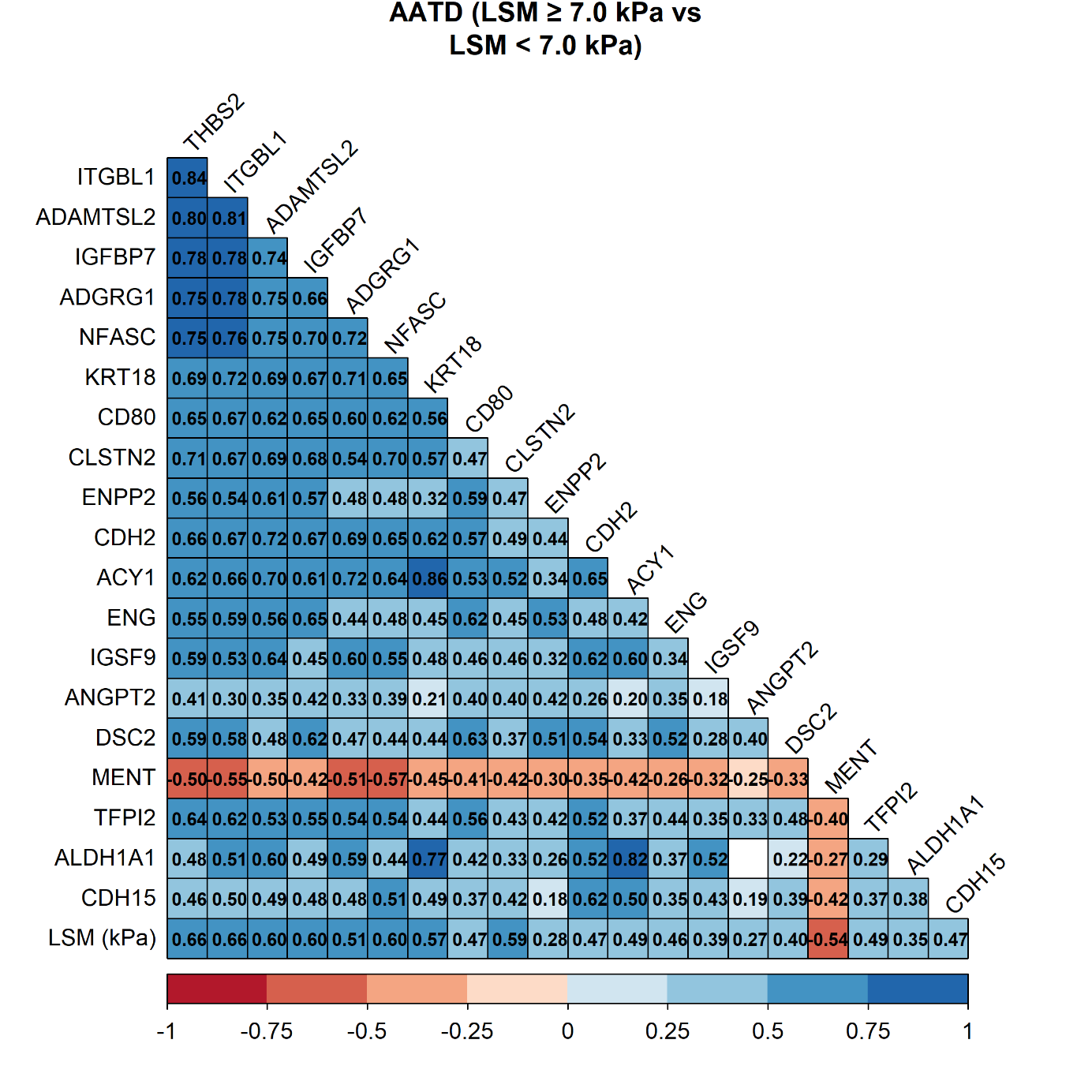


**Fig. S6: Correlation of 20 biomarkers in the cohort of alpha1-antitrypsin deficiency (AATD) subjects.** Displayed are the Spearman rank correlation coefficients. Liver fibrosis was assessed with non-invasive liver stiffness measurement (LSM, reported in kPa, via FibroScan®). *ACY1: aminoacylase 1; ADAMTSL2: ADAMTS-like protein 2; ADGRG1: adhesion G protein-coupled receptor G1; ALDH1A1: aldehyde dehydrogenase 1A1; ANGPT2: angiopoietin 2; CD80: CD80 molecule; CDH2: cadherin 2; CDH15: cadherin 15; CLSTN2: calsyntenin 2; DSC2: desmocollin 2; ENG: endoglin; ENPP2: ectonucleotide pyrophosphatase/phosphodiesterase 2; IGFBP7: insulin-like growth factor-binding protein 7; IGSF9: Immunoglobulin superfamily member 9; ITGBL1: integrin beta-like protein 1; KRT18: keratin-18; MENT: C1orf56 (chromosome 1 open reading frame 56); NFASC: neurofascin; TFPI2: tissue factor pathway inhibitor 2; THBS2: thrombospondin-2.*


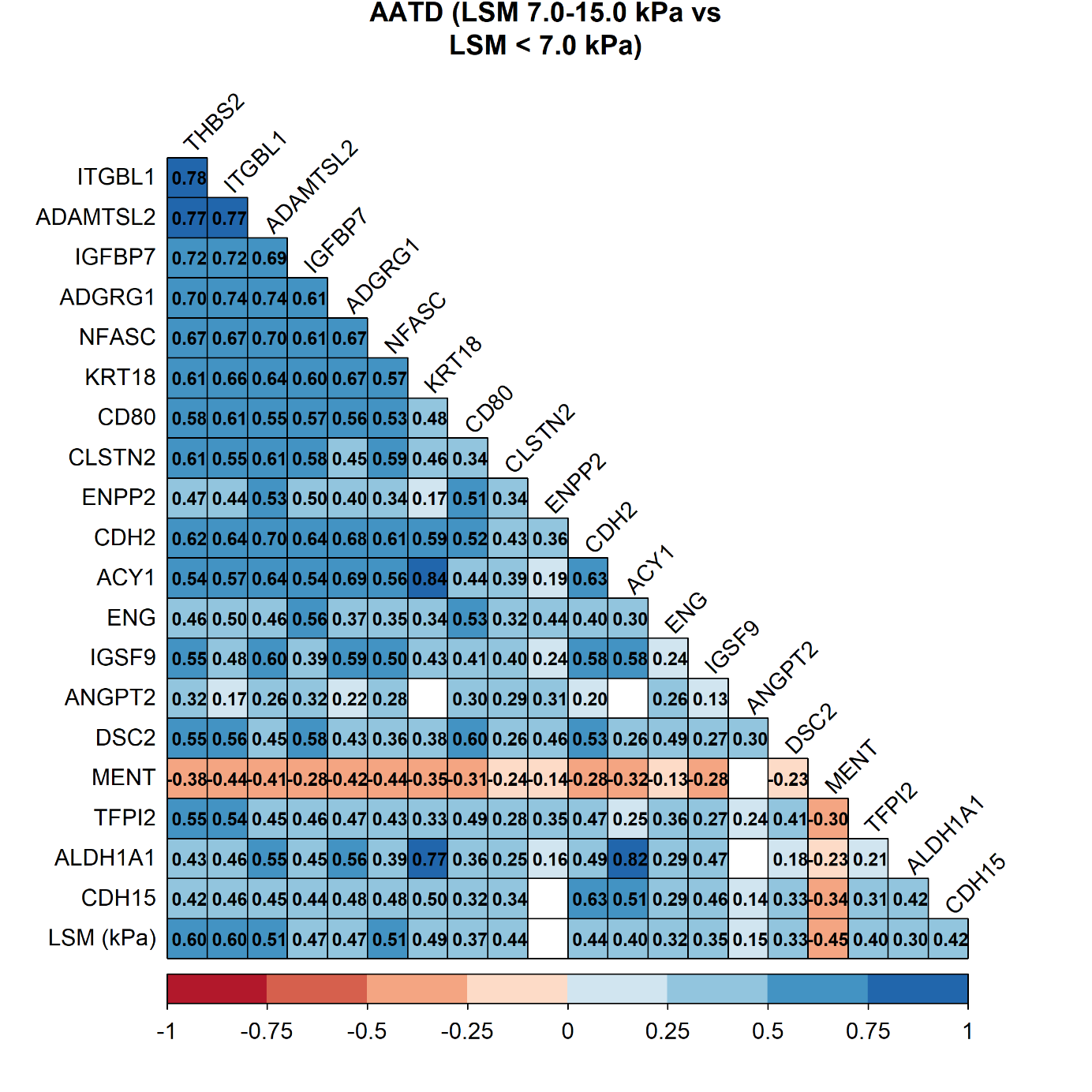


**Fig. S7: Correlation of 20 biomarkers in a subcohort of alpha1-antitrypsin deficiency (AATD) subjects without possible clinically significant portal hypertension.** Displayed are the Spearman rank correlation coefficients. Liver fibrosis was assessed with non-invasive liver stiffness measurement (LSM, reported in kPa, via FibroScan®) and subjects with LSM ≥15kPa were excluded. *ACY1: aminoacylase 1; ADAMTSL2: ADAMTS-like protein 2; ADGRG1: adhesion G protein-coupled receptor G1; ALDH1A1: aldehyde dehydrogenase 1A1; ANGPT2: angiopoietin 2; CD80: CD80 molecule; CDH2: cadherin 2; CDH15: cadherin 15; CLSTN2: calsyntenin 2; DSC2: desmocollin 2; ENG: endoglin; ENPP2: ectonucleotide pyrophosphatase/phosphodiesterase 2; IGFBP7: insulin-like growth factor-binding protein 7; IGSF9: Immunoglobulin superfamily member 9; ITGBL1: integrin beta-like protein 1; KRT18: keratin-18; MENT: C1orf56 (chromosome 1 open reading frame 56); NFASC: neurofascin; TFPI2: tissue factor pathway inhibitor 2; THBS2: thrombospondin-2.*


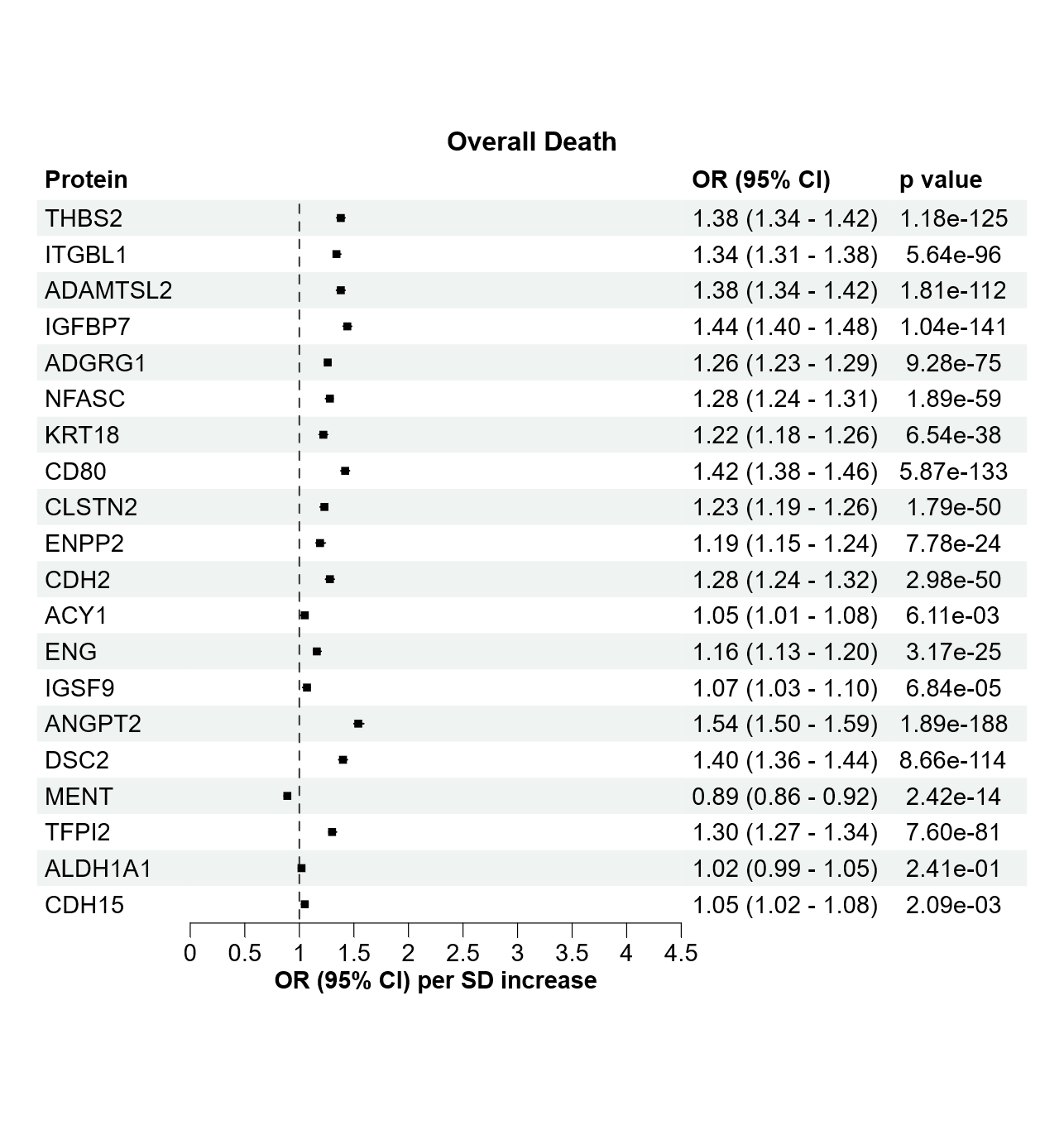


**Fig. S8. Association of overall mortality with plasma levels of selected proteomic biomarkers in the UK Biobank (UKB).** Odds ratios (OR) and *P* values were generated from logistic regression models (adjusted for age, sex, and BMI). OR (95% CI) per standard deviation increase of plasma levels are shown. *ACY1: aminoacylase 1; ADAMTSL2: ADAMTS-like protein 2; ADGRG1: adhesion G protein-coupled receptor G1; ALDH1A1: aldehyde dehydrogenase 1A1; ANGPT2: angiopoietin 2; CD80: CD80 molecule; CDH2: cadherin 2; CDH15: cadherin 15; CLSTN2: calsyntenin 2; DSC2: desmocollin 2; ENG: endoglin; ENPP2: ectonucleotide pyrophosphatase/phosphodiesterase 2; IGFBP7: insulin-like growth factor-binding protein 7; IGSF9: Immunoglobulin superfamily member 9; ITGBL1: integrin beta-like protein 1; KRT18: keratin-18; MENT: C1orf56 (chromosome 1 open reading frame 56); NFASC: neurofascin; TFPI2: tissue factor pathway inhibitor 2; THBS2: thrombospondin-2.*


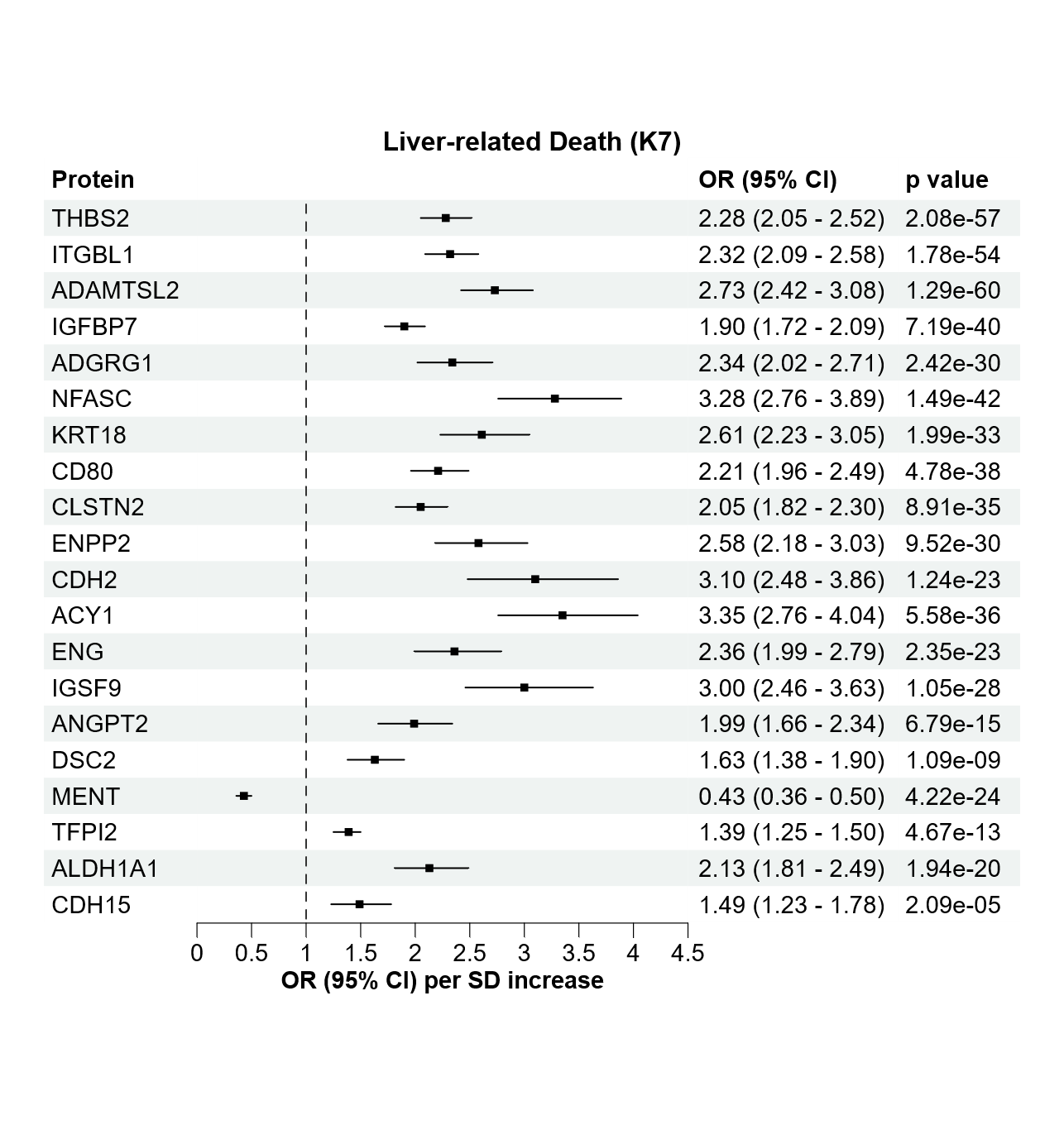
**Fig. S9. Association of liver-related mortality with plasma levels of selected proteomic biomarkers in the UK Biobank (UKB).** Odds ratios (OR) and *P* values were generated from logistic regression models (adjusted for age, sex, and BMI). OR (95% CI) per standard deviation increase of plasma levels are shown. Liver-related mortality was defined via primary causes of death related to ICD-10 diagnosis codes K70 to K77. *ACY1: aminoacylase 1; ADAMTSL2: ADAMTS-like protein 2; ADGRG1: adhesion G protein-coupled receptor G1; ALDH1A1: aldehyde dehydrogenase 1A1; ANGPT2: angiopoietin 2; CD80: CD80 molecule; CDH2: cadherin 2; CDH15: cadherin 15; CLSTN2: calsyntenin 2; DSC2: desmocollin 2; ENG: endoglin; ENPP2: ectonucleotide pyrophosphatase/phosphodiesterase 2; ICD-10: International Classification of Diseases version 10;* *IGFBP7: insulin-like growth factor-binding protein 7; IGSF9: Immunoglobulin superfamily member 9; ITGBL1: integrin beta-like protein 1; KRT18: keratin-18; MENT: C1orf56 (chromosome 1 open reading frame 56); NFASC: neurofascin; TFPI2: tissue factor pathway inhibitor 2; THBS2: thrombospondin-2.*


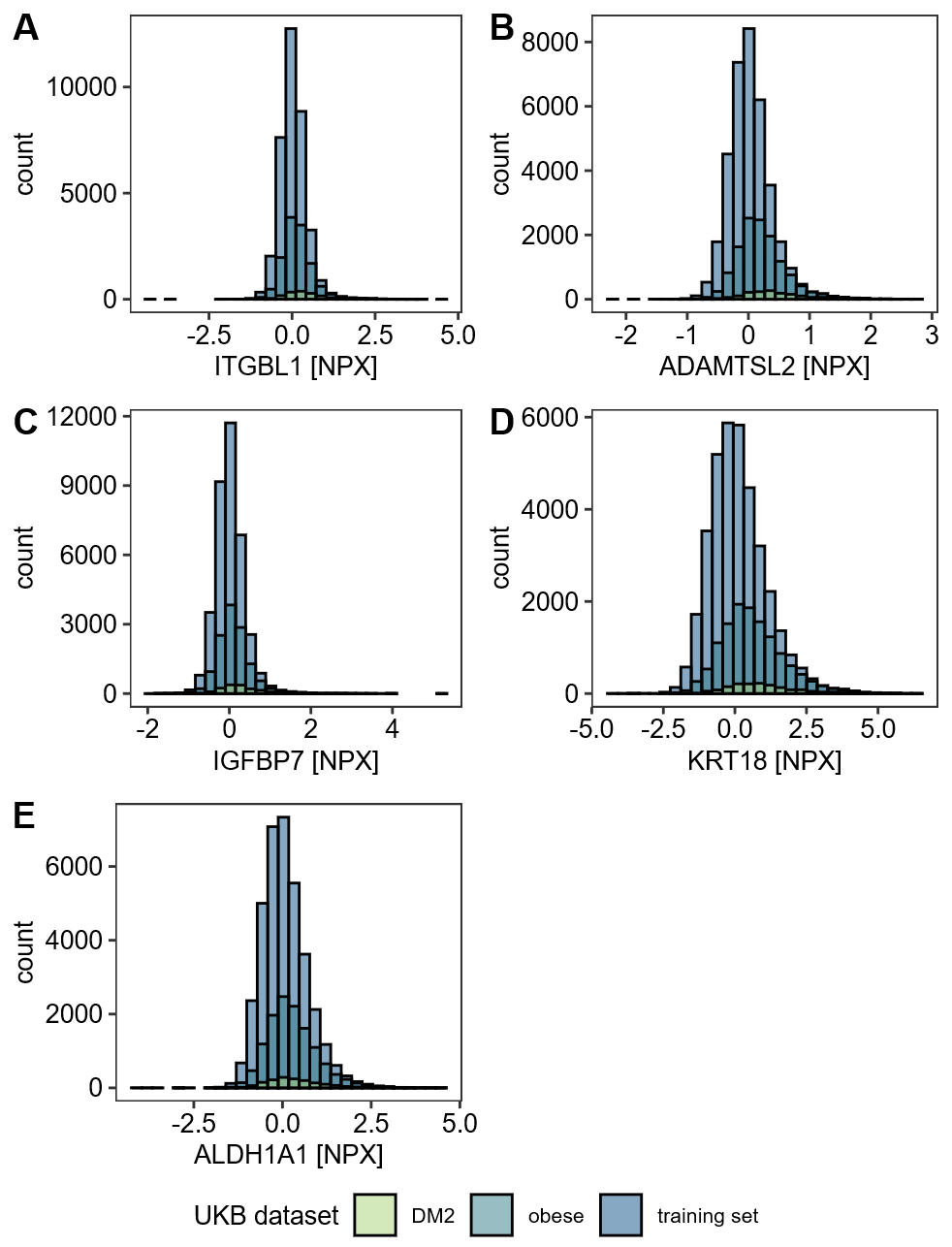


**Fig. S10: Distribution of continuous parameters assessed in multivariable logistic regression during development of PEA score.** Histograms display the distribution of the levels of the depicted five parameters in the UK Biobank (UKB) training set (N=36,399), as well as two subcohorts of obese participants (BMI >30 kg/m², N=12,735) and those with type 2 diabetes mellitus (DM2, N=1,577) (x-axis, normalised expression values [NPX]). A: integrin beta-like protein 1 [ITGBL1]; B: ADAMTS-like protein 2 [ADAMTSL2]; C: insulin-like growth factor-binding protein 7 [IGFBP7]; D: keratin-18 [KRT18]; E: aldehyde dehydrogenase 1A1 [ALDH1A1]. *DM2: type-2 diabetes; PEA: proximity extension assay.*


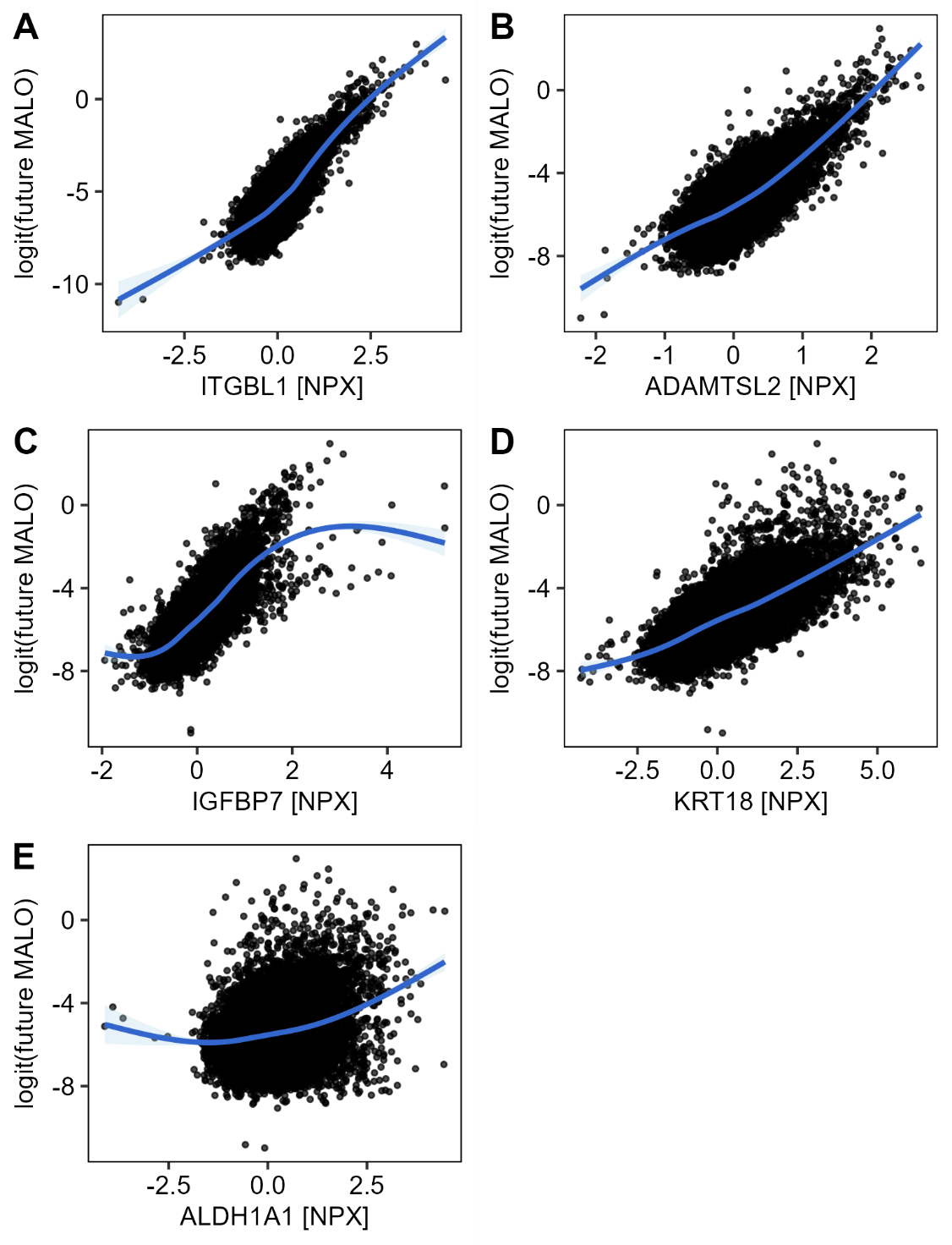


**Fig. S11: The relationship between parameters in the PEA score and the logit of the development of a future major adverse liver outcome (MALO).** Scatter plots visualise the relationship between the parameters chosen for the PEA score in the UK Biobank training set (N=36,399) (x-axis, normalised expression values [NPX]) and the logit of a future MALO (y-axis). A: integrin beta-like protein 1 [ITGBL1]; B: ADAMTS-like protein 2 [ADAMTSL2]; C: insulin-like growth factor-binding protein 7 [IGFBP7]; D: keratin-18 [KRT18]; E: aldehyde dehydrogenase 1A1 [ALDH1A1]. *PEA: proximity extension assay.*


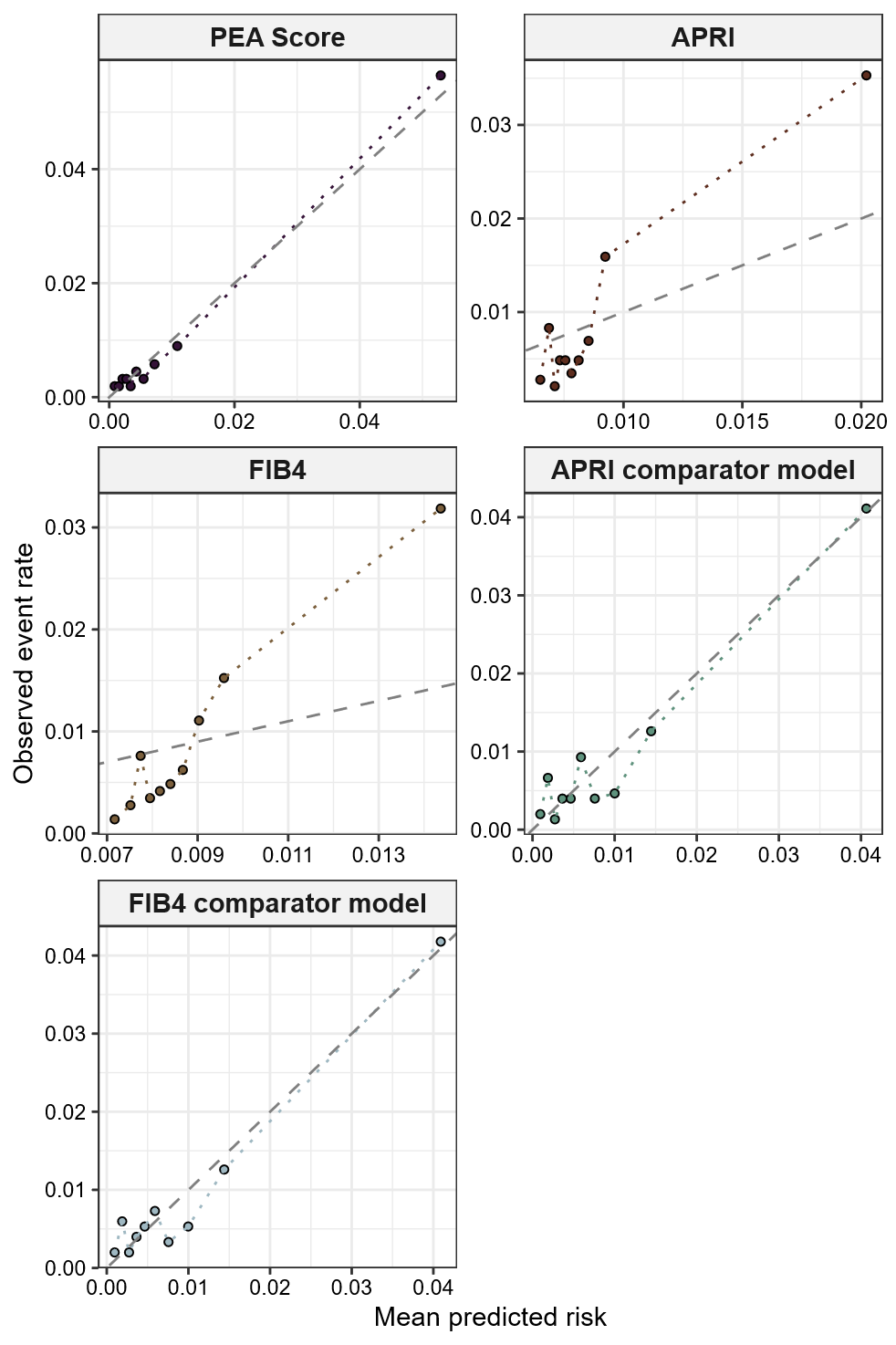


**Figure S12: Calibration of logistic regression models for the prediction of major adverse liver outcomes (MALOs) in the UK Biobank test set.** Each panel displays the mean predicted risk (x-axis) against the observed event rate (y-axis) across the deciles of predicted risk for the PEA score, APRI, FIB4, as well as the APRI- and FIB4-comparator models. The dashed diagonal line represents perfect calibration. Axes are scaled to the observed range of predicted risk to enable visualisation of lower deciles. Analyses were performed in the 30% test set (N=15,599). *APRI: AST-to-platelet-ratio index; FIB4: Fibrosis-4 index*


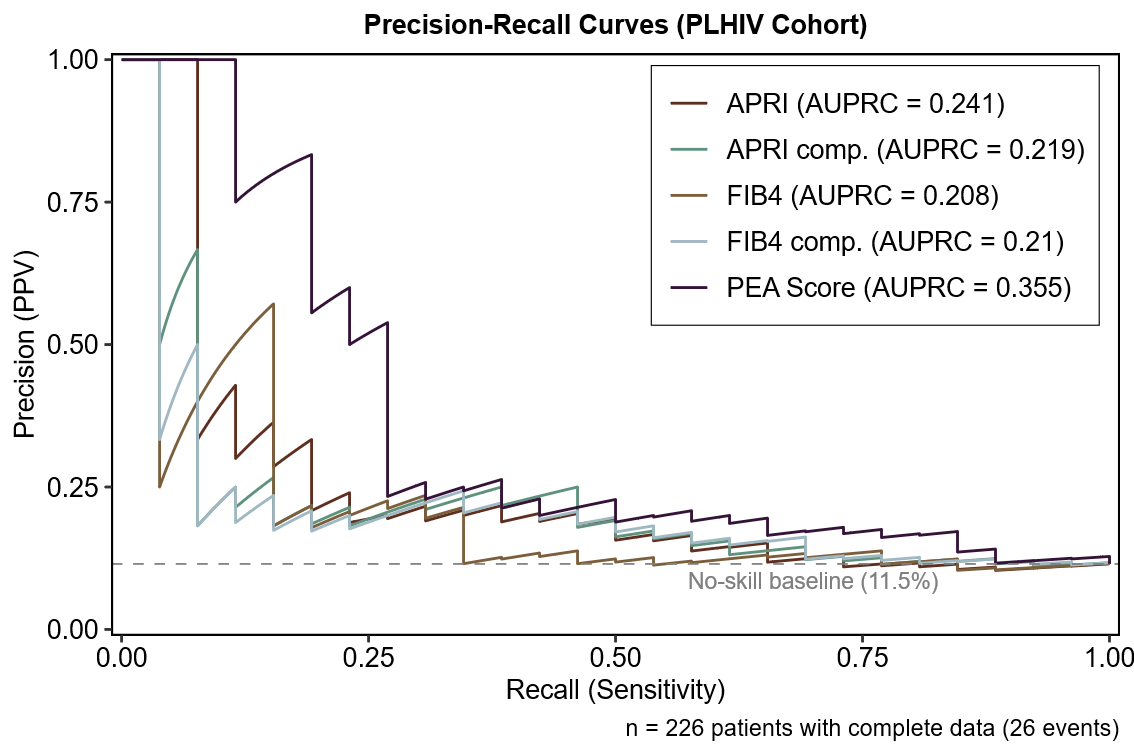


**Figure S13: Precision-recall curves for the PEA score and established clinical indices in the PLHIV cohort.** Precision-recall curves for the PEA score, APRI, FIB4, as well as the APRI- and FIB4-comparator models in the subset of PLHIV patients with complete available data for all tested models (N=226, 26 events/cases with LSM ≥7.0 kPa). The dashed horizontal line represents the no-skill baseline, corresponding to the prevalence of LSM≥7.0 kPa in this subset (~11.5%). The area under the precision-recall curves (AUPRC) is presented for each model. *APRI: AST-to-platelet-ratio index; FIB4: Fibrosis-4 index; LSM: liver stiffness measurements; PLHIV: people living with HIV.*


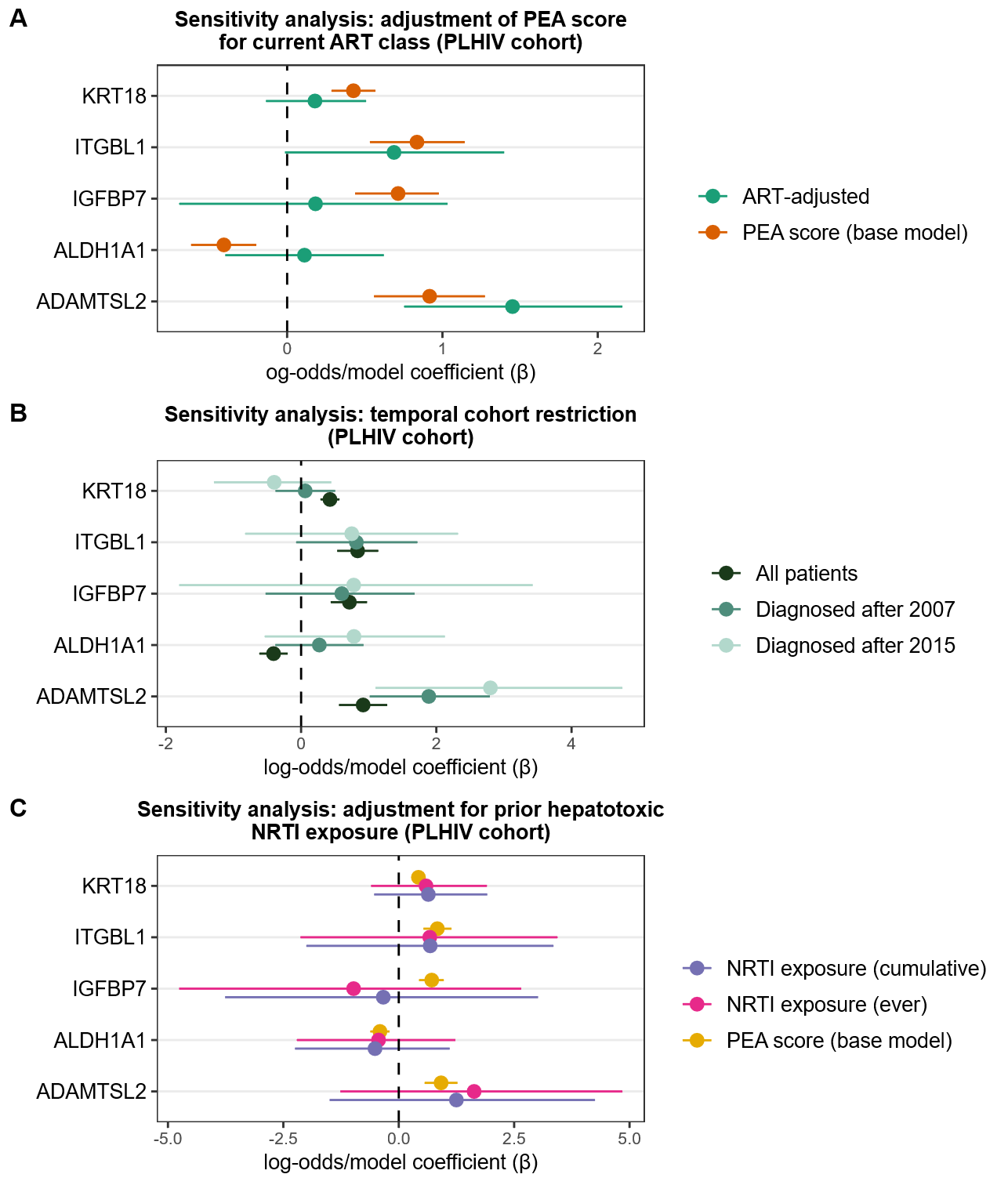


**Figure S14: Sensitivity analyses assessing the robustness of the PEA score to antiretroviral therapy-related cofounding in the PLHIV cohort.** Log-odds estimates (β) with corresponding 95% confidence intervals are shown for each of the five proteins comprising the PEA score. A: Comparison of protein coefficients between the unadjusted base model and a model additionally adjusted for current antiretroviral therapy (ART) class (non-nucleoside reverse transcriptase inhibitors [NNRTI], integrase strand transfer inhibitors [INSTI] or protease inhibitors [PI]). B: Protein coefficients across the full cohort and two temporally restricted cohorts (including only patients who were diagnosed after 2007 and after 2015, respectively. C: Comparison of protein coefficients between the unadjusted base model and models additionally adjusted for prior exposure to hepatotoxic nucleoside reverse transcriptase inhibitors (NRTIs), modelled both binary (ever vs never exposed) and as a cumulative duration. All models included age and sex as covariates. *ADAMTSL2: ADAMTS-like protein 2; ALDH1A1: aldehyde dehydrogenase 1A1; IGFBP7: insulin-like growth factor-binding protein 7; ITGBL1: integrin beta-like protein 1; KRT18: keratin-18.*


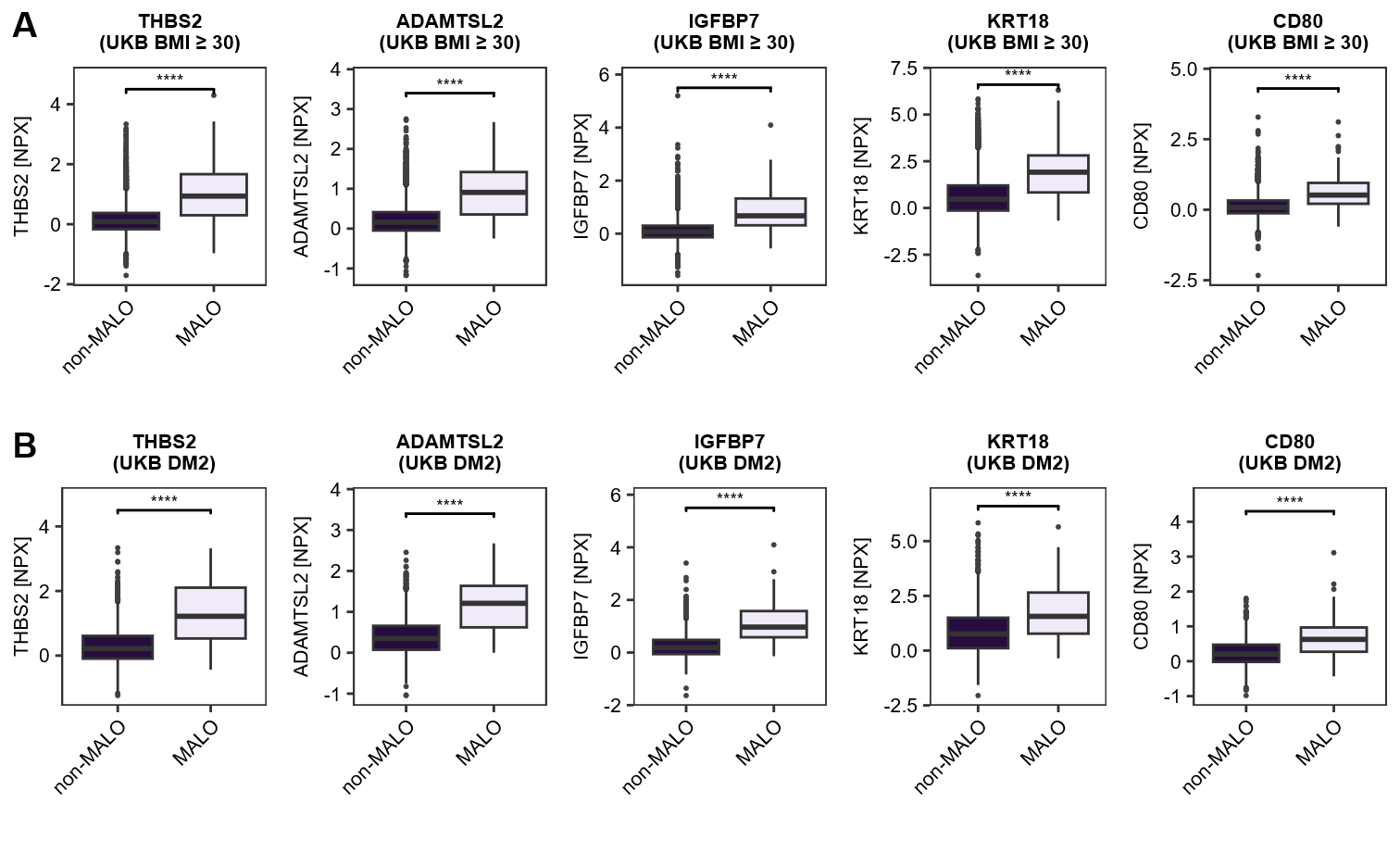


**Fig. S15:** **Levels of selected proteomic markers in obese UK Biobank (UKB) participants and those with type 2 diabetes mellitus subdivided based on the development of major adverse liver outcomes (MALOs).** Box plots display relative expression levels (NPX values) of five protein markers (ITGBL1, ADAMTSL2, IGFBP7, KRT18, and ALDH1A1) in subgroups of UKB subjects with A: obesity (BMI ≥30 kg/m²) and B: type-2 diabetes (DM2), comparing subjects with versus those without MALOs. A Wilcoxon rank sum test was used to calculate *P* values. Significance levels are indicated as follows: ns: not significant, **p* <0·05, ***p* <0·01, ****p* <0·001, *****p* <0·0001. *ADAMTSL2: ADAMTS-like protein 2; ALDH1A1: aldehyde dehydrogenase 1A1; IGFBP7: insulin-like growth factor-binding protein 7; ITGBL1: integrin beta-like protein 1; KRT18: keratin-18.*

**
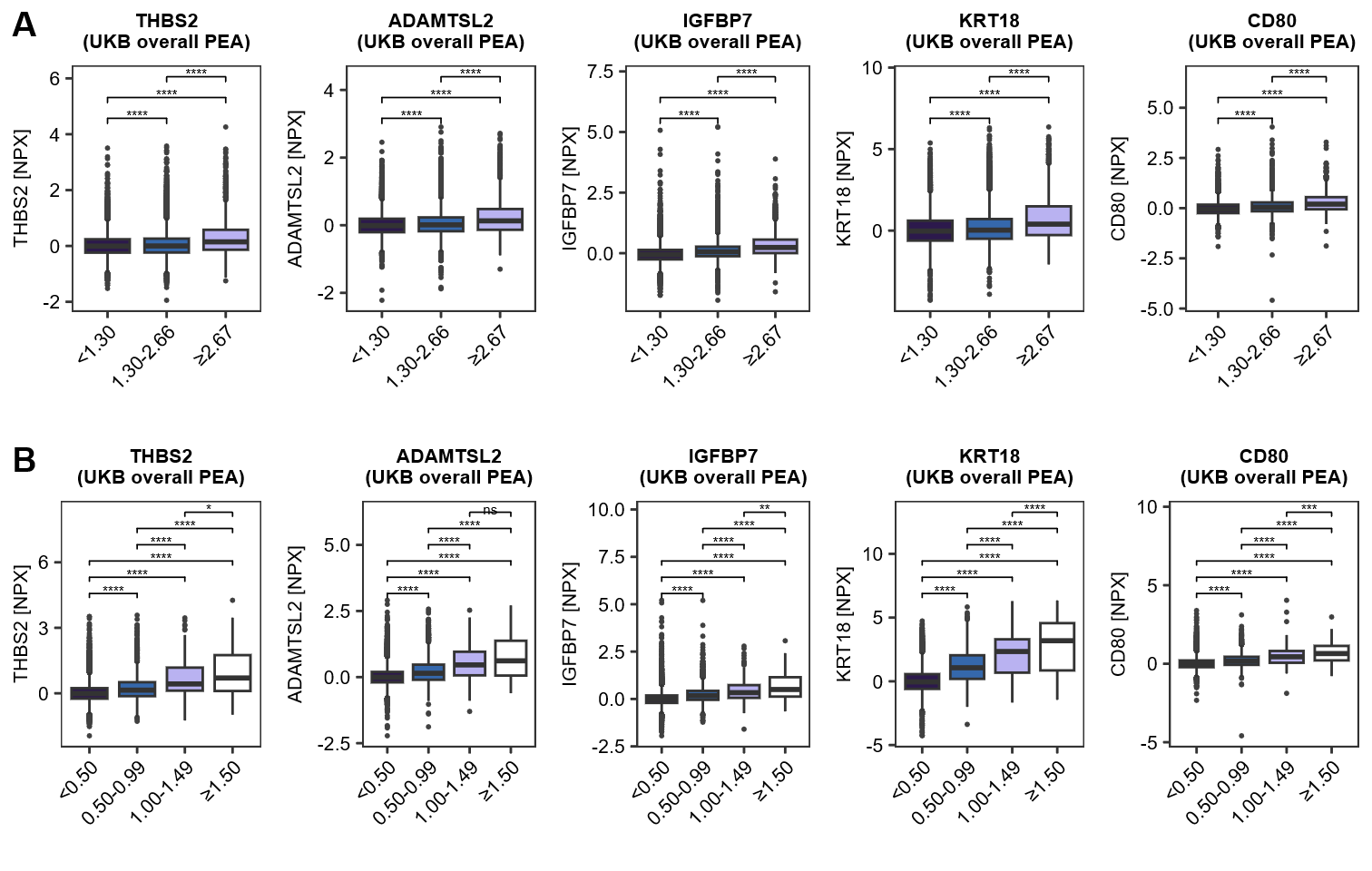
**

**Fig. S16: Ability of selected biomarkers to discriminate between liver fibrosis stages in the UK Biobank (UKB) cohort.** Box plots display relative expression levels (NPX values) of five protein markers (ITGBL1, ADAMTSL2, IGFBP7, KRT18, and ALDH1A1) in the UKB population with available proximity extension assay (PEA) proteomic data. They compare subjects from different liver fibrosis severity stages, stratified via non-invasive liver fibrosis surrogates. A: Fibrosis-4 (FIB4) and B: AST-to-platelet ratio index (APRI). A Wilcoxon rank sum test was used to compare subgroups. Significance levels are indicated as follows: ns: not significant, **p* <0·05, ***p* <0·01, ****p* <0·001, *****p* <0·0001. *ADAMTSL2: ADAMTS-like protein 2; ALDH1A1: aldehyde dehydrogenase 1A1; IGFBP7: insulin-like growth factor-binding protein 7; ITGBL1: integrin beta-like protein 1; KRT18: keratin-18.*


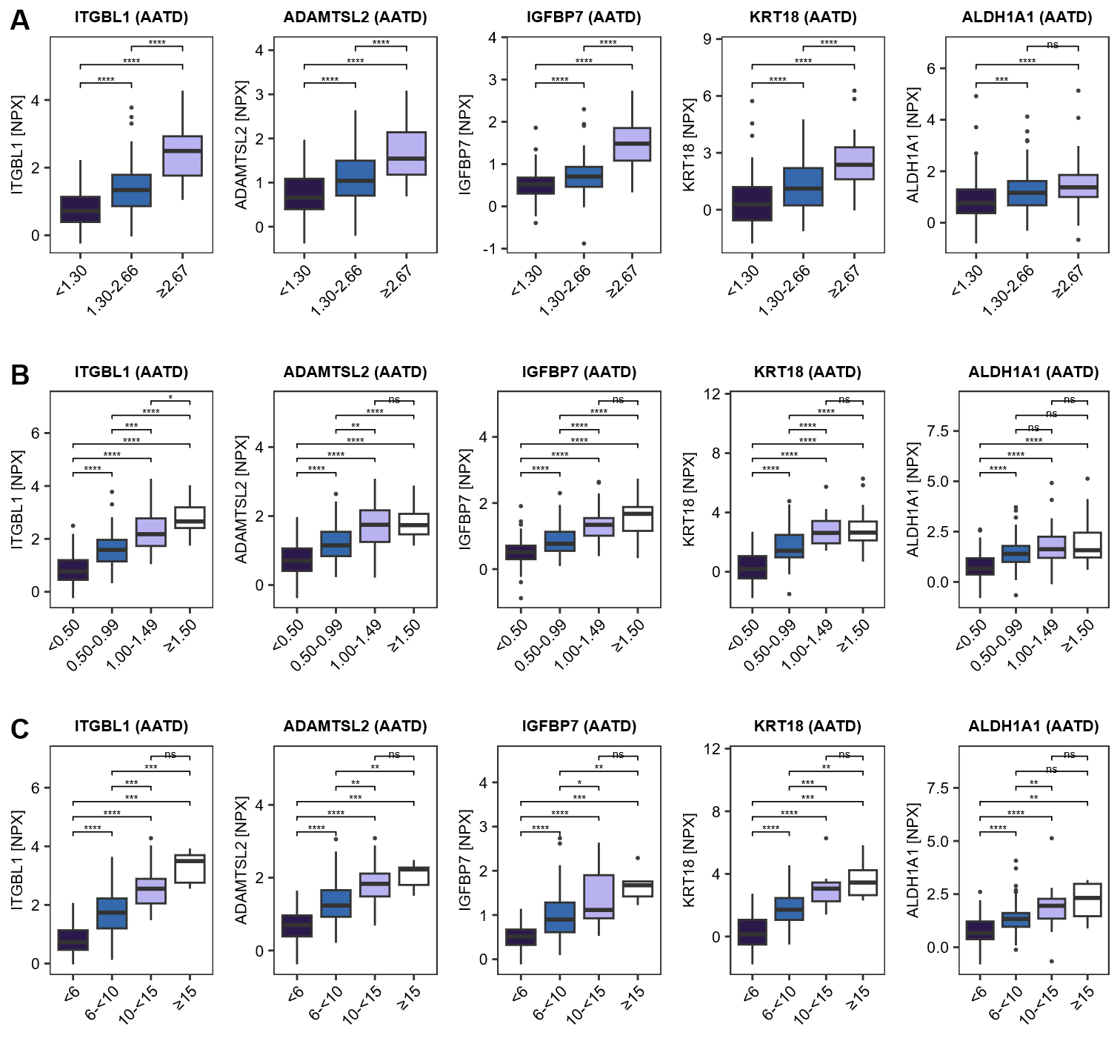


**Fig. S17: Ability of selected biomarkers to discriminate between liver fibrosis stages in a cohort of severe alpha1-antitrypsin deficiency (AATD) subjects.** Box plots display relative expression levels (NPX values) of five protein markers (ITGBL1, ADAMTSL2, IGFBP7, KRT18, and ALDH1A1) in the entire AATD cohort. They compare patients with different liver fibrosis severity stages, stratified via non-invasive liver fibrosis surrogates. A: Fibrosis-4 (FIB4), B: AST-to-platelet ratio index (APRI) and C: LiverRisk score. A Wilcoxon rank sum test was used to compare individual subgroups. Significance levels are indicated as follows: ns: not significant, **p* <0·05, ***p* <0·01, ****p* <0·001, *****p* <0·0001. *ADAMTSL2: ADAMTS-like protein 2; ALDH1A1: aldehyde dehydrogenase 1A1; IGFBP7: insulin-like growth factor-binding protein 7; ITGBL1: integrin beta-like protein 1; KRT18: keratin-18.*

# Supplementary Tables

**Table S1. List of ICD-10 and OPCS-4 codes.** Below are the ICD-10 and OPCS-4 codes used in this study. Codes marked with ***** are ascites codes that were only considered in combination with at least one chronic liver disease ICD-10 code (ICD-10: K70-K77). This assignment was used because ascites can have non-hepatic causes. ICD-10: Z944 was used to identify patients who received a liver transplant prior to baseline assessment and were excluded from the study. *ICD-10: International Classification of Diseases version 10, Tenth Revision; OPCS-4: operation/procedure codes version 4.*

| **List of ICD-10 codes used** | **Description** |
| --- | --- |
| K70.3 | Alcoholic cirrhosis of liver |
| K72.1 | Chronic hepatic failure |
| K74.6 | Other and unspecified cirrhosis of liver |
| K76.6 | Portal hypertension |
| K76.7 | Hepatorenal syndrome |
| I85.0; I859; I98.2; I98.3 | Oesophageal varices |
| I86.4 | Gastric varices |
| C22.0 | Hepatocellular carcinoma |
| Z94.4 | Liver transplant status |
|  | |
| **List of OPCS-4 codes used** | **Description** |
| J06.1 | Transjugular intrahepatic insertion of stent into portal vein |
| J06.2 | Transjugular intrahepatic insertion of stent graft into portal vein |
| G10.4 | Local ligation of varices of oesophagus |
| G10.8 | Other specified open operations on varices of oesophagus |
| G10.9 | Unspecified open operations on varices of oesophagus |
| G14.4 | Fiberoptic endoscopic injection sclerotherapy to varices of oesophagus |
| G17.4 | Endoscopic injection sclerotherapy to varices of oesophagus using rigid oesophagoscope |
| G43.7 | Fiberoptic endoscopic rubber band ligation of upper gastrointestinal tract varices |
| T46.1* | Paracentesis abdominis for ascites |
| T46.2* | Drainage of ascites not elsewhere specified |

**Table S2. Demographics and clinical parameters of obese participants (BMI>30 kg/m²) with available proteomic data from the UK Biobank cohort.** Cohorts of participants who did vs. did not develop major adverse liver outcomes during the follow-up (MALO/non-MALO) are shown. Data are expressed as median (25^th^-75^th^ percentile) for continuous variables and n (%) for categorical variables. P-values for continuous variables were obtained from linear regression analyses († without covariates, ‡ with covariates age, sex, and BMI). Associations between categorical variables were assessed using Fisher’s exact test. Parameters with *p* <1·00E-100 are highlighted in bold. *ALT: alanine aminotransferase; ALP: alkaline phosphatase; AST: aspartate aminotransferase; GGT* *gamma-glutamyltransferase;* *HbA1C: haemoglobin A1c.*

| **Characteristics** | **Non-MALOs**, n=12,522 | **MALOs**, n=213 | ***p* value** |
| --- | --- | --- | --- |
| Age | 59 (51-64) | 62 (56-65) | 1.15E-06† |
| Sex (Female) | 6,657 (53%) | 68 (32%) | 6.75E-10 |
| BMI (kg/m^2^) | 32.8 (31.2-35.5) | 33.5 (31.8-36.9) | 4.25E-05† |
| Type-2 Diabetes | 876 (7.0%) | 47 (22%) | 3.33E-12 |
| Blood glucose (mmol/L) | 5.03 (4.67-5.53) | 5.34 (4.79-6.77) | 7.75E-12‡ |
| HbA1C (mmol/mol) | 37 (34-40) | 39 (34-48) | 1.96E-04‡ |
| Lipid-lowering medication | 3,201 (26%) | 81 (38%) | 7.15E-05 |
| Total protein (g/L) | 72.4 (69.9-75.2) | 73.0 (70.1-76.6) | 2.19E-03‡ |
| Albumin (g/L) | 44.57 (42.85-46.28) | 43.33 (41.58-45.34) | 6.05E-13‡ |
| ALT (IU/L) | 24 (18-33) | 32 (21-51) | 2.15E-34‡ |
| AST (IU/L) | 25 (21-30) | 36 (27-52) | 2.04E-97‡ |
| **GGT (IU/L)** | **34 (23-51)** | **90 (49-198)** | **4.81E-189‡** |
| ALP (IU/L) | 86 (72-101) | 94 (80-121) | 3.22E-30‡ |
| Total bilirubin (µmol/L) | 7.7 (6.1-9.9) | 9.3 (6.9-12.8) | 2.44E-19‡ |
| Direct bilirubin (µmol/L) | 1.59 (1.29-2.06) | 2.16 (1.54-3.10) | 2.40E-60‡ |
| Platelets (10^9^/L) | 252 (216-292) | 210 (162-254) | 1.10E-17‡ |

**Table S3. Demographics and clinical parameters of participants with type-2 diabetes with available proteomic data from the UK Biobank cohort.** Cohorts of participants who did vs. did not develop major adverse liver outcomes during the follow-up (MALO/non-MALO) are shown. Data are expressed as median (25^th^-75^th^ percentile) for continuous variables and n (%) for categorical variables. P-values for continuous variables were obtained from linear regression analyses († without covariates, ‡ with covariates age, sex, and BMI). Associations between categorical variables were assessed using Fisher’s exact test. *ALT: alanine aminotransferase; ALP: alkaline phosphatase; AST: aspartate aminotransferase; GGT: gamma-glutamyltransferase; HbA1C: haemoglobin A1c.*

| **Characteristics** | **Non-MALOs**, n=1,507 | **MALOs**, n=70 | ***p* value** |
| --- | --- | --- | --- |
| Age | 62 (57-66) | 64 (61-67) | 2.02E-03† |
| Sex (Female) | 562 (37%) | 23 (33%) | 0.527† |
| BMI (kg/m^2^) | 31.3 (27.5-35.2) | 32.4 (28.9-35.5) | 0.242† |
| Blood glucose (mmol/L) | 6.5 (5.3-8.8) | 6.9 (5.4-9.4) | 0.04‡ |
| HbA1C (mmol/mol) | 50 (43-59) | 51 (42-60) | 0.823‡ |
| Lipid-lowering medication | 1,128 (75%) | 41 (59%) | 0.005 |
| Total protein (g/L) | 72.4 (69.5-75.4) | 73.9 (69.9-77.1) | 0.015‡ |
| Albumin (g/L) | 44.8 (42.7-46.7) | 43.4 (40.2-45.6) | 1.08E-04‡ |
| ALT (IU/L) | 24 (18-34) | 28 (17-43) | 6.70E-05‡ |
| AST (IU/L) | 25 (21-31) | 33 (26-43) | 6.34E-14‡ |
| GGT (IU/L) | 35 (23-54) | 75 (45-183) | 1.12E-23‡ |
| ALP (IU/L) | 86 (71-103) | 96 (79-128) | 1.93E-04‡ |
| Total bilirubin (µmol/L) | 7.8 (6.1-10.1) | 8.7 (6.9-13.1) | 9.61E-08‡ |
| Direct bilirubin (µmol/L) | 1.74 (1.38-2.33) | 2.17 (1.54-3.23) | 2.13E-09‡ |
| Platelets (10^9^/L) | 242 (206-292) | 207 (157-251) | 2.48E-07‡ |

**Table S4. Differential abundance analysis (Bayesian linear regression) comparing participants from the UK Biobank cohort with available proximity extension assay (PEA) data with/without future major adverse liver outcomes (MALO).** A log2 fold-change >0 indicates proteins elevated in participants with vs. without future MALO. 1748 proteins differed between both groups (FDR <0·05, 1325 elevated, 4423 diminished in MALO). Data are sorted according to FDR. Tissue-specificities are indicated with a 0/1-coding (1: specificity for a given tissue). *logFC: log fold change; p: p value; FDR: false discovery rate.*

**Table S5. Differential abundance analysis (Bayesian linear regression) comparing obese (BMI ≥30 kg/m²) participants from the UK Biobank with available proximity extension assay (PEA) data with/without future major adverse liver outcomes (MALO).** A log2 fold-change >0 indicates proteins elevated in participants with vs. without future MALO. 1703 proteins differed between both groups (FDR <0·05, 1165 elevated, 538 diminished in MALO). Data are sorted according to FDR. Tissue-specificities are indicated with a 0/1-coding (1: specificity for a given tissue). *logFC: log fold change; p: p value; FDR: false discovery rate.*

**Table S6. Differential abundance analysis (Bayesian linear regression) comparing Type-2 diabetic (DM2) participants from the UK Biobank with available proximity extension assay (PEA) data with/without future major adverse liver outcomes (MALO).** A log2 fold-change >0 indicates proteins elevated in participants with vs. without future MALO. 817 proteins differed between both groups (FDR <0·05, 758 elevated, 59 diminished in MALO). Data are sorted according to FDR. Tissue-specificities are indicated with a 0/1-coding (1: specificity for a given tissue). *logFC: log fold change; p: p value; FDR: false discovery rate.*

**Table S7. Differential abundance analysis (Bayesian linear regression) comparing AATD patients with vs. without significant liver fibrosis based on non-invasive liver stiffness measurement (LSM ≥7·0 kPa vs. <7·0 kPa).** A log2 fold-change >0 indicates proteins elevated in patients with LSM ≥7·0 kPa vs. <7·0 kPa. 1266 proteins differed between both groups (FDR <0·05, 792 elevated, 470 diminished in subjects with LSM ≥7·0 kPa). Data are sorted according to FDR. Tissue-specificities are indicated with a 0/1-coding (1: specificity for a given tissue). *AATD: alpha1-antitrypsin deficiency; logFC: log fold change; p: p value; FDR: false discovery rate.*

**Table S8. Differential abundance analysis (Bayesian linear regression) comparing AATD patients with vs. without significant liver fibrosis based on non-invasive liver stiffness measurement (LSM 7·0-<15kPa vs. <7·0 kPa).** A log2 fold-change > 0 indicates proteins elevated in patients with LSM of 7·0-<15kPa vs. <7·0 kPa. Subjects with possible clinically significant portal hypertension (LSM≥15) were excluded. 548 proteins differed between both groups (FDR <0.05, 442 elevated, 106 diminished in subjects with LSM of 7·0-<15kPa). Data are sorted according to FDR. Tissue-specificities are indicated with a 0/1-coding (1: specificity for a given tissue). *AATD: alpha1-antitrypsin deficiency; logFC: log fold change; p: p value; FDR: false discovery rate.*

**Table S9. Demographic and routine parameters of people living with HIV (PLHIV).** Categorisation of subjects was based on non-invasive liver stiffness measurement (LSM), values are expressed in kPa. Data are expressed as median (25^th^-75^th^ percentile) for continuous variables and n (%) for categorical variables. P-values for continuous variables were obtained from linear regression analyses († without covariates, ‡ with covariates age, sex, and BMI). Associations between categorical variables were assessed using Fisher’s exact test. *ALT: alanine aminotransferase; ALP: alkaline phosphatase; AST: aspartate aminotransferase; GGT: gamma-glutamyltransferase.*

| **Characteristics** | **n** | **PLHIV cohort, Fibrosis grade based on LSM (kPa)** | | ***p* value** |
| --- | --- | --- | --- | --- |
|  |  | **F0-F1**  (<7·0)  n=874 | **≥F2**  (≥7·0)  n=86 |  |
| Age (years) | 960 | 52 (43-59) | 54 (41-62) | 0.541† |
| Sex (Female) | 960 | 120 (14%) | 8 (9.3%) | 0.318 |
| BMI (kg/m^2^) | 960 | 24.9 (22.5-27.5) | 27.6 (24.3-30.9) | 8.54E-09† |
| Type-2 Diabetes | 960 | 38 (4.3%) | 13 (15%) | 2.75E-04‡ |
| ALT (IU/L) | 939 | 25 (20-33) | 31 (21-43) | 1.50E-06‡ |
| AST (IU/L) | 227 | 26 (21-31) | 28 (22-36) | 0.022‡ |
| GGT (IU/L) | 386 | 28 (18-49) | 33 (24-68) | 0.082‡ |
| ALP (IU/L) | 413 | 79 (66-94) | 82 (66-99) | 0.258‡ |
| Total bilirubin (µmol/L) | 768 | 7.0 (5.0-10.0) | 7.0 (6.0-10.0) | 0.022‡ |
| Platelets (10^9^/L) | 957 | 216 (186-256) | 224 (183-275) | 0.128‡ |

**Table S10. Ability of selected biomarkers to predict MALOs** **in the UK Biobank (UKB) cohort.** The table displays areas under receiving operating curves (AUROCs) for the 20 proteins consistently associated with a significant liver disease in all studied cohorts (UKB, AATD and PLHIV) for the prediction of future major adverse liver outcomes (MALO) (logistic regression, age and sex added as covariates). AUROCs were calculated for all UKB participants with available proximity extension assay (PEA) data allocated to the test set (30%), as well as the obese (BMI ≥30 kg/m²) and diabetic (DM2) subgroups. Variables are sorted in descending order of AUROC in the UKB test set. *AATD: alpha1-antitrypsin deficiency; ACY1: aminoacylase 1; ADAMTSL2: ADAMTS-like protein 2; ADGRG1: adhesion G protein-coupled receptor G1; ALDH1A1: aldehyde dehydrogenase 1A1; ALT: alanine aminotransferase; ANGPT2: angiopoietin 2; AST: aspartate aminotransferase; CD80: CD80 molecule; CDH2: cadherin 2; CDH15: cadherin 15; CLSTN2: calsyntenin 2; DSC2: desmocollin 2; ENG: endoglin; ENPP2: ectonucleotide pyrophosphatase/phosphodiesterase 2; GGT: gamma-glutamyltransferase; IGF1: insulin-like growth factor 1; IGFBP7: insulin-like growth factor-binding protein 7; IGSF9: Immunoglobulin superfamily member 9; ITGBL1: integrin beta-like protein 1; KRT18: keratin-18; MENT: C1orf56 (chromosome 1 open reading frame 56); NFASC: neurofascin; PLHIV: people living with HIV; TFPI2: tissue factor pathway inhibitor 2; THBS2: thrombospondin-2.*

|  | **AUROCs** | | | **Absolute differences in AUROCs** | |
| --- | --- | --- | --- | --- | --- |
|  | **UKB test set** | **Obese** | **DM2** | **\|UKB test set -Obese\|** | **\|UKB test set - DM2\|** |
| **ITGBL1** | 0.807 | 0.837 | 0.865 | 0.030 | 0.057 |
| **ADAMTSL2** | 0.803 | 0.833 | 0.819 | 0.030 | 0.016 |
| **IGFBP7** | 0.799 | 0.835 | 0.847 | 0.036 | 0.049 |
| **THBS2** | 0.795 | 0.832 | 0.834 | 0.036 | 0.039 |
| **NFASC** | 0.794 | 0.812 | 0.799 | 0.018 | 0.005 |
| **GGT (baseline, clinical chemistry)** | 0.788 | 0.820 | 0.794 | 0.032 | 0.006 |
| **ADGRG1** | 0.777 | 0.799 | 0.781 | 0.021 | 0.004 |
| **CDH2** | 0.774 | 0.791 | 0.769 | 0.017 | 0.005 |
| **CD80** | 0.767 | 0.806 | 0.748 | 0.039 | 0.019 |
| **KRT18** | 0.764 | 0.790 | 0.714 | 0.026 | 0.050 |
| **ENPP2** | 0.750 | 0.785 | 0.769 | 0.036 | 0.019 |
| **ACY1** | 0.748 | 0.763 | 0.695 | 0.015 | 0.053 |
| **CLSTN2** | 0.748 | 0.797 | 0.812 | 0.050 | 0.064 |
| **IGF1 (baseline, clinical chemistry)** | 0.744 | 0.767 | 0.749 | 0.023 | 0.005 |
| **AST (baseline, clinical chemistry)** | 0.741 | 0.773 | 0.731 | 0.032 | 0.010 |
| **ENG** | 0.740 | 0.773 | 0.755 | 0.034 | 0.015 |
| **IGSF9** | 0.729 | 0.755 | 0.732 | 0.025 | 0.002 |
| **MENT** | 0.715 | 0.756 | 0.727 | 0.040 | 0.012 |
| **ANGPT2** | 0.713 | 0.746 | 0.679 | 0.032 | 0.034 |
| **DSC2** | 0.708 | 0.772 | 0.725 | 0.064 | 0.017 |
| **ALDH1A1** | 0.706 | 0.734 | 0.677 | 0.029 | 0.028 |
| **TFPI2** | 0.686 | 0.730 | 0.682 | 0.044 | 0.003 |
| **ALT (baseline, clinical chemistry)** | 0.677 | 0.711 | 0.613 | 0.034 | 0.064 |
| **CDH15** | 0.633 | 0.694 | 0.649 | 0.061 | 0.016 |

**Table S12: Performance of top-ranked four-, five-, and six-feature proteomic models in the UK biobank cohort.** All possible combinations of four, five and six proteins from the 20 candidate proteins consistently associated with liver disease across all assessed cohorts were systematically evaluated. All models additionally included age and sex as covariates, but these are not listed in the “Variables“ column. The top five models per feature category are shown. For further consideration, candidate models were required to meet pre-specified quality criteria (all coefficients p<0.05, maximum pairwise correlation ≤0.7, VIFs < 0.5). The selected five-feature PEA score (comprising ITGBL1, ADAMTSL2, IGFBP7, KRT18, ALDH1A1) is highlighted in bold and was chosen based on the principle of parsimony and its consistent discriminative performance across all assessed cohorts. AUROC values are reported for the UKB training and test set, as well as the subcohort of obese patients (BMI≥30 kg/m²) and those with type 2 diabetes mellitus (DM2). *AUC: area under the receiving operator characteristic curve; VIF: variance inflation factor.*

| **N (Features)** | **Variables** | **AUC**  **(train)** | **max. corr** | **p** | **VIF** | **AUC**  **(test)** | **AUC**  **(obese)** | **AUC**  **DM2)** |
| --- | --- | --- | --- | --- | --- | --- | --- | --- |
| **4** | IGFBP7  ADGRG1  KRT18  ANGPT2 | 0.829 | 0.388 | <2.2E-16  4.8E-14  4.7E-14  1.6E-04 | 1.516  1.792  1.467  1.371 | 0.838 | 0.859 | 0.840 |
| **4** | IGFBP7  ADGRG1  ACY1  ANGPT2 | 0.825 | 0.382 | <2.2E-16  5.9E-15  1.7E-11  3.2E-05 | 1.44  1.797  1.491  1.354 | 0.837 | 0.854 | 0.843 |
| **4** | IGFBP7  ADGRG1  ANGPT2  ALDH1A1 | 0.818 | 0.382 | <2.2E-16  <2.2E-16  5.1E-05  8.6E-02 | 1.456  1.66  1.373  1.302 | 0.838 | 0.852 | 0.849 |
| **4** | IGFBP7  ADGRG1  ANGPT2  CDH15 | 0.817 | 0.382 | <2.2E-16  <2.2E-16  9.1E-05  0.627 | 1.447  1.537  1.387  1.425 | 0.832 | 0.846 | 0.844 |
| **4** | IGFBP7  NFASC  ADGRG1  KRT18 | 0.824 | 0.496 | <2.2E-16  2.0E-14  5.9E-05  1.63E-06 | 1.519  2.383  2.68  1.758 | 0.835 | 0.853 | 0.849 |
| **5** | IGFBP7  ADGRG1  KRT18  ANGPT2  CDH15 | 0.830 | 0.388 | <2.2E-16  5.6E-14  4.4E-14  1.4E-04  0.560 | 1.516  1.860  1.598  1.377  1.446 | 0.838 | 0.860 | 0.840 |
| **5** | IGFBP7  ADGRG1  ACY1  ANGPT2  CDH15 | 0.826 | 0.382 | <2.2E-16  1.4E-14  1.8E-11  3.1E-05  0.795 | 1.451  1.878  1.507  1.361  1.491 | 0.837 | 0.855 | 0.842 |
| **5** | IGFBP7  ADGRG1  ANGPT2  ALDH1A1  CDH15 | 0.817 | 0.382 | <2.2E-16  <2.2E-16  5.9E-05  9.4E-03  0.766 | 1.159  1.761  1.380  1.309  1.425 | 0.837 | 0.851 | 0.848 |
| **5** | IGFBP7  NFASC  ADGRG1  KRT18  ANGPT2 | 0.827 | 0.496 | <2.2E-16  2.4E-12  9.9E-05  9.3E-07  0.03 | 1.704  2.491  2.155  1.748  1.467 | 0.837 | 0.854 | 0.847 |
| **5**  **(PEA score)** | **ITGBL1**  **ADAMTSL2**  **IGFBP7**  **KRT18**  **ALDH1A1** | **0.837** | **0.467** | **8.2E-08**  **5.0E-07**  **2.0E-07**  **3.7E-07**  **1.5E-04** | **3.952**  **3.80**  **2.108**  **3.033**  **2.615** | **0.827** | **0.859** | **0.854** |
| **6** | ADAMTSL2  IGFBP7  ADGRG1  KRT18  CDH2  CDH15 | 0.832 | 0.470 | 1.3E-12  1.3E-15  4.9E-05  4.9E-04  0.051  0.182 | 2.88  1.715  2.1695  1.987  2.099  1.496 | 0.858 | 0.860 | 0.860 |
| **6** | ADAMTSL2  IGFBP7  THBS2  CD80  KRT18  ALDH1A1 | 0.833 | 0.581 | 3.4E-05  6.8E-08  2.9E-02  2.5E-03  1.5E-09  1.1E-05 | 4.067  1.969  3.653  2.147  1.569  3.155 | 0.857 | 0.860 | 0.857 |
| **6** | ADAMTSL2  IGFBP7  THBS2  ADGRG1  KRT18  CDH15 | 0.831 | 0.581 | 1.1E-05  1.8E-12  7.3E-04  1.8E-03  3.4E-03  0.362 | 4.18  1.85  3.70  2.217  1.923  1.441 | 0.859 | 0.861 | 0.864 |
| **6** | ADAMTSL2  IGFBP7  THBS2  ADGRG1  KRT18  TFPI2 | 0.828 | 0.581 | 1.5E-05  2.2E-11  1.4E-02  2.7E-03  5.1E-03  0.345 | 4.154  1.917  3.799  2.169  1.908  1.330 | 0.860 | 0.858 | 0.865 |
| **6** | ADAMTSL2  IGFBP7  THBS2  KRT18  ALDH1A1  TFPI2 | 0.829 | 0.581 | 4.1E-09  1.2E-10  8.3E-05  1.7E-09  2.6E-05  0.474 | 3.706  1.909  3.692  3.033  2.652  1.345 | 0.857 | 0.858 | 0.859 |

**Table S13: Performance metrics of the PEA score and established clinical indices at the Youden-optimal threshold in the PLHIV cohort.** Sensitivity, specificity, positive predictive value (PPV) and negative predictive value (NPV) were calculated at the Youden-optimal threshold for each model (PEA score, APRI, FIB4, as well as the APRI- and FIB4-comparator models) separately. Analyses were restricted to patients with complete data for all scores/indices (N= 226, 26 events/cases with LSM ≥7.0 kPa). *APRI: AST-to-platelet-ratio index; FIB4: Fibrosis-4 index; PLHIV: people living with HIV.*

| **Model** | **Threshold** | **Sensitivity** | **Specificity** | **PPV** | **NPV** |
| --- | --- | --- | --- | --- | --- |
| PEA score | 0.006 | 0.846 | 0.470 | 0.172 | 0.959 |
| APRI | 0.388 | 0.500 | 0.730 | 0.194 | 0.918 |
| FIB4 | 1.734 | 0.346 | 0.835 | 0.214 | 0.908 |
| APRI comp. | 1.000 | 0.462 | 0.820 | 0.250 | 0.921 |
| FIB4 comp. | 1.000 | 0.423 | 0.815 | 0.229 | 0.916 |

**Table S14: Protein coefficients across sensitivity analyses in the PLHIV cohort.** Log-odds estimates (β) with 95% confidence intervals (CIs) are shown for each of the five proteins comprising the PEA score across six model specifications: the unadjusted base model (PEA score), a model adjusted for current classes of antiretroviral therapy (ART), two temporally restricted cohorts (patients diagnoses before 2007 and before 2015) and two models adjusting for prior exposure to hepatotoxic nucleoside reverse transcriptase inhibitors (NRTIs). All models additionally included age and sex as covariates. *ADAMTSL2: ADAMTS-like protein 2; ALDH1A1: aldehyde dehydrogenase 1A1; IGFBP7: insulin-like growth factor-binding protein 7; ITGBL1: integrin beta-like protein 1; KRT18: keratin-18.*

| **term** | **Base model (PEA score) β (95% CI)** | **ART-adjusted**  **β (95% CI)** | **Diagnosis**  **post 2007**  **β (95% CI)** | **Diagnosis**  **post 2015**  **β (95% CI)** | **NRTI ever**  **β (95% CI)** | **NRTI cumulative**  **β (95% CI)** |
| --- | --- | --- | --- | --- | --- | --- |
| **ITGBL1** | 0.84  (0.53-1.14) | 0.69  (-0.01-1.40) | 0.82  (-0.07-1.72) | 0.75  (-0.83-2.32) | 0.68  (-2.00-3.35) | 0.67  (-2.13-3.44) |
| **ADAMTSL2** | 0.92  (0.56-1.27) | 1.45  (0.75-2.16) | 1.89  (1.01-2.79) | 2.80  (1.10-4.75) | 1.25  (-1.50-4.25) | 1.63  (-1.27-4.85) |
| **IGFBP7** | 0.71  (0.44-0.98) | 0.18  (-0.70-1.03) | 0.60  (-0.53-1.68) | 0.78  (-1.80-3.43) | -0.34  (-3.76-3.02) | -0.98  (-4.76-2.65) |
| **KRT18** | 0.43  (0.29-0.57) | 0.18  (-0.14-0.51) | 0.06  (-0.38-0.51) | -0.40  (-1.29-0.45) | 0.64  (-0.53-1.92) | 0.59  (-0.60-1.91) |
| **ALDH1A1** | -0.41  (-0.62--0.20) | 0.11  (-0.40-0.62) | 0.27  (-0.38-0.92) | 0.78  (-0.54-2.13) | -0.51  (-2.25-1.11) | -0.44  (-2.21-1.23) |
| **AUROC** | 0.69 (0.63-0.75) | 0.74 (0.68-0.80) | 0.73 (0.67-0.79) | 0.71 (0.64-0.78) | 0.67 (0.61-0.74) | 0.73 (0.55-0.91) |
